# Effectiveness of guided internet-delivered cognitive behavioral therapy for insomnia in autistic adults (i-Sleep Autism): a randomized controlled trial

**DOI:** 10.1093/sleep/zsag199

**Published:** 2026-07-19

**Authors:** Kirsten L Spaargaren, Sander Begeer, Smiddy Nieuwenhuis, Jessie Bradley, Heleen Riper, Kirstin Greaves-Lord, Annemieke van Straten

**Affiliations:** Department of Clinical, Neuro-, and Developmental Psychology, Vrije Universiteit Amsterdam, Amsterdam, the Netherlands; Mental Health, Amsterdam Public Health Research Institute, Amsterdam, the Netherlands; Department of Clinical, Neuro-, and Developmental Psychology, Vrije Universiteit Amsterdam, Amsterdam, the Netherlands; Mental Health, Amsterdam Public Health Research Institute, Amsterdam, the Netherlands; Department of Clinical, Neuro-, and Developmental Psychology, Vrije Universiteit Amsterdam, Amsterdam, the Netherlands; LEARN! Research Institute, Vrije Universiteit, Amsterdam, the Netherlands; Policy Analysis section of the faculty of Technology, Policy and Management, Delft University of Technology, Delft, the Netherlands; Department of Clinical, Neuro-, and Developmental Psychology, Vrije Universiteit Amsterdam, Amsterdam, the Netherlands; Mental Health, Amsterdam Public Health Research Institute, Amsterdam, the Netherlands; Department of Psychiatry, Amsterdam University Medical Centre, Vrije Universiteit, Amsterdam, the Netherlands; Department of Psychology, Clinical Psychology and Experimental Psychopathology Unit, University of Groningen, Groningen, the Netherlands; Jonx, Department of (Youth) Mental Health and Autism, Autism Team Northern-Netherlands, Lentis Psychiatric Institute, Groningen, the Netherlands; Department of Clinical, Neuro-, and Developmental Psychology, Vrije Universiteit Amsterdam, Amsterdam, the Netherlands; Mental Health, Amsterdam Public Health Research Institute, Amsterdam, the Netherlands

**Keywords:** autism, adults, insomnia, internet-delivered CBT-I, RCT, sleep intervention, participatory action research

## Abstract

An estimated 60% of autistic adults suffer from insomnia symptoms, which significantly impairs daily functioning and wellbeing. This study evaluated the effectiveness of i-Sleep Autism, a 5-week, guided self-help internet-delivered cognitive behavioral therapy-based intervention (ICBT-I) for insomnia in autistic adults. In co-creation with seven autistic adults with sleeping problems, i-Sleep Autism was adapted from i-Sleep, a pre-existing non-autism specific intervention. A total of 163 adults with autism and insomnia (ISI ≥ 10) was randomly allocated to i-Sleep Autism (n = 82) or waitlist control (n = 81; receiving psychoeducation and sleep hygiene). Participants were assessed at baseline (T0), mid-intervention (T1; 3-weeks), post-test (T2; 6-weeks), and follow-up (T3; 6 months) on insomnia severity (primary outcome), pre-sleep arousal, anxiety, depression, daily functioning, and quality of life. In the intervention group, 56.1% completed four, and 50% all five modules. Intent-To-Treat analyses revealed a significant reduction in insomnia severity at T1 and T2 (*d* = 1.02, 95%CI = [0.65, 1.40]), and cognitive- and somatic pre-sleep arousal at post-test, when comparing intervention to control. The intervention group showed significantly higher quality of life at post-test. At T2, 37.8% of the intervention group versus 11.1% of the control group showed clinically meaningful improvement of insomnia. Effects on insomnia severity were maintained at 6-month follow up. No significant effects were found for anxiety, depression, or daily functioning. This is the first RCT to investigate autism-adapted ICBT-I for autistic adults suffering from insomnia. i-Sleep Autism effectively and sustainably improves insomnia. Additional strategies are needed to support dropouts and those who did not benefit.

**Statement of Significance**

Insomnia is very common among autistic adults and has substantial consequences for daily functioning and well-being, yet evidence-based sleep interventions for this group are lacking. This study provides the first randomized controlled evaluation of an internet-delivered cognitive behavioral intervention for insomnia that was adapted in collaboration with autistic adults. The findings demonstrate that autism-adapted, guided, digital sleep interventions can meaningfully improve sleep and related outcomes in autistic adults, with effects that are sustained over time. At the same time, considerable variation in engagement and response highlights the need for further refinement and personalization. Future research should focus on identifying who benefits most, optimizing support for non-responders, and improving accessibility and adherence.

## Graphical Abstract

**Graphical Abstract:**
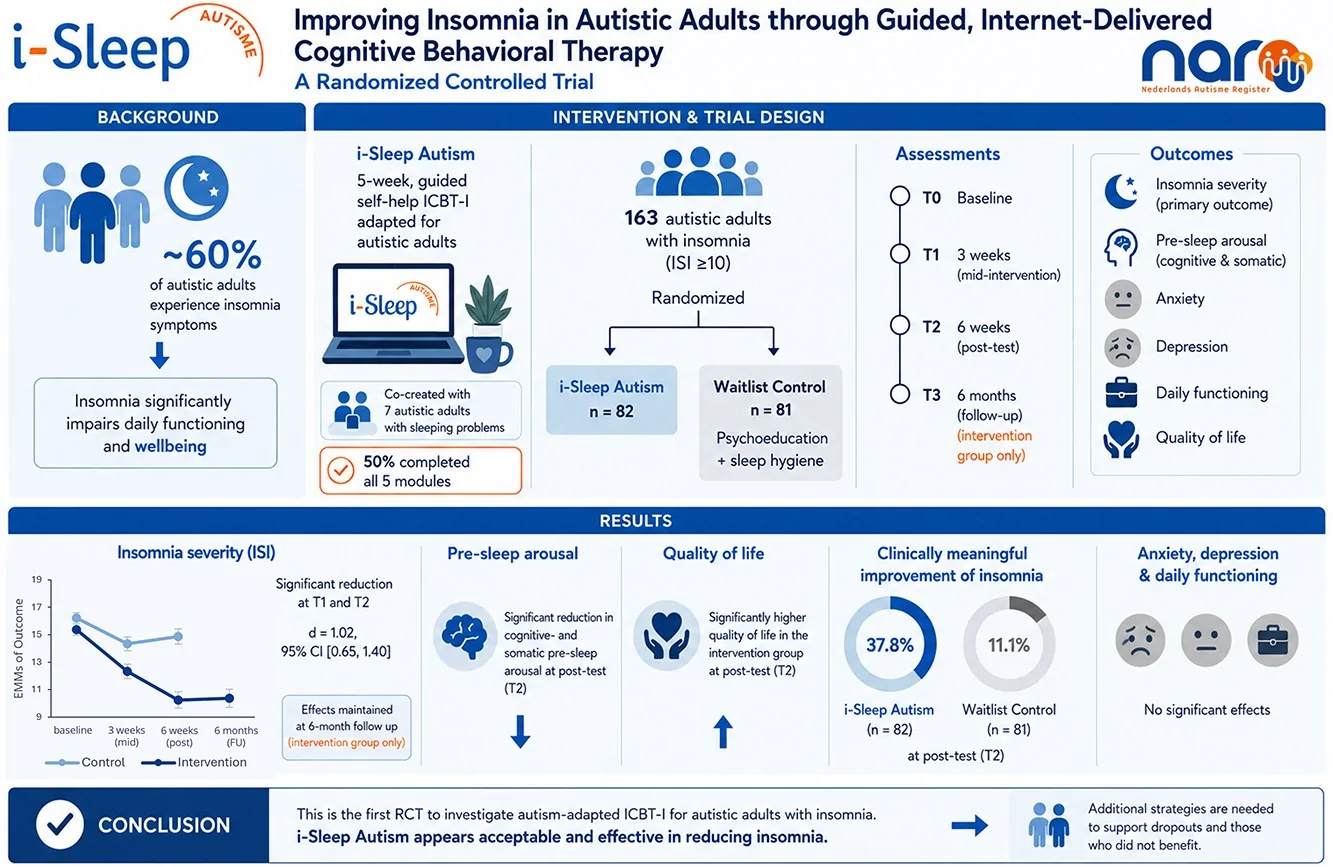

## Introduction

Sleep problems, particularly insomnia, are highly prevalent among adults with autism spectrum disorder (autism from hereon^1^), affecting up to 79%–89% [2–9]. Autism occurs in approximately 1% of the global population [10] and is defined by distinctive patterns in social interaction, communication, sensory processing, and focused interests, which can be both strengths and sources of challenge [11, 12]. An estimated 60% of autistic individuals is affected by insomnia symptoms specifically [4], implying difficulties falling asleep, staying asleep, and waking up early without being able to fall back asleep and causing significant daytime impairment. Sleeping problems such as insomnia reduce quality of life [13, 14], and have been associated with depression [15], suicidality [16], more pronounced characteristics of autism, and poorer psychosocial, executive, and physical functioning [17]. Considering the high prevalence of sleep problems and the substantial impact on wellbeing, it is not surprising that sleep is ranked among the top priorities for research and practice identified by the autistic community [18].

Autism and insomnia may be linked in reinforcing, bidirectional feedback loops, where sleep problems and autistic characteristics exacerbate one another [17, 19], although literature is inconclusive [20]. The widely used 3P Behavioral model by Spielman, Caruso and Glovinsky [21] posits that insomnia arises from an interaction of predisposing (e.g. biological or psychological vulnerabilities), precipitating (e.g. life events, stress), and perpetuating factors (e.g. maladaptive sleep habits or cognitions). For autism, predisposing factors may include sensory processing differences [22, 23], increased anxiety and stress [24–26], female sex at birth, co-occurring attention deficit hyperactivity disorder (ADHD) or other mental health conditions, and physical health problems (e.g. gastrointestinal problems) [27–29]. Precipitating factors may include the more frequent occurrence of stressful life events in autistic individuals compared to non-autistic individuals [25], and disruptions of routines through intolerance of uncertainty and preference for predictability [30, 31]. Perpetuating factors may include hyperarousal [32, 33], and lack of daily structure (e.g. through unemployment, common in autism) [34]. Overall, most predisposing factors for insomnia symptoms in autism appear similar to those in non-autistic populations.

In the general population, The European guidelines on insomnia recommend cognitive behavioral therapy for insomnia (CBT-I) as the first-line treatment [35]. CBT-I is an umbrella term for structured, nonpharmacological treatment employing educational, cognitive and behavioral strategies to improve insomnia, for instance including cognitive restructuring, sleep restriction and stimulus control [36]. A comprehensive body of research has demonstrated the benefits of CBT-I, with large effect sizes and long-term effectiveness [37–39]. CBT-I is also effective in reducing insomnia severity of patients with co-occurring mental disorders, including depression, post-traumatic stress disorder (PTSD), and alcohol dependency, and is shown to simultaneously improve the symptom severity of these conditions [40]. These results underscore the robustness of CBT-I’s effectiveness in improving insomnia, suggesting this may extend to autistic individuals.

However, the effectiveness of CBT-I for autistic adults remains unclear to date. Psychological interventions for insomnia in autism, including CBT-I, have sparsely been part of scientific investigation, with most existing studies focusing on youth populations [41–43]. These interventions have shown promising effects on sleep outcomes and daytime behaviors in autistic youth [41, 44]. A recent meta-analysis (11 studies, 850 participants) found a large effect of behavioral interventions on the subjective Children’s Sleep Habits Questionnaire (*d* = 1.01), and a small but significant improvement of objective measures total sleep time (TST) (*d* = 0.31), reduction of sleep onset latency (*d* = 0.35) [44]. To date, no randomized controlled trials have evaluated CBT-I specifically for autistic adults [42, 43]. A small pilot study (*n* = 10) into the effectiveness of a 5-week transdiagnostic group treatment of adults with autism and/or ADHD focusing on sleep education, scheduling, hygiene, and relaxation yielded improvement of sleep disturbances [45], but this intervention did not include sleep restriction nor cognitive restructuring. Another pilot study (*n* = 8) in adults indicated that a group-based insomnia intervention using Acceptance and Commitment Therapy, a third-wave therapy, was effective in reducing both insomnia and anxiety symptomatology [46].

Generally, given the limited availability of resources to provide such psychological interventions, pharmacological interventions are frequently prescribed for insomnia [47]. However, many licensed sleep medications offer short-term effectiveness but are poorly tolerated and risk negative side effects and addiction. In autism, melatonin is among the most used agents to address sleep difficulties and has shown benefits for some sleep outcomes, including TST, sleep latency and sleep efficiency [2]. Other pharmacological interventions are also frequently prescribed to autistic individuals for sleep problems, despite a lack of research on their effectiveness in this population [48, 49], and despite autistic adults’ preference for non-medicament treatments such as education and advice [49].

Like in non-autistic populations, only a minority of autistic adults with insomnia seek help [49]. Based on their experiences, they have reported limited faith in existing treatments and emphasize the need for participatory research co-creating interventions “sensitive to autistic experiences” [50]. Co-creation may be particularly important given that differences in sensory and information processing, communication styles, and preferences for predictability and routine can influence how interventions are experienced. For example, core CBT-I components such as sleep restriction may be challenging because they require changes to established routines [51]. Core characteristics of autism may further pose a barrier in (accessing) traditional healthcare. For example, difficulties with eye contact, decoding body language and simultaneously processing different perceptual modalities tend to make face-to-face contact with healthcare professionals strenuous [52]. Together with the limited service availability, these findings highlight the need for more scalable, non-pharmacological sleep interventions, developed in collaboration with those they are intended to serve.

Guided, internet-delivered CBT-I (ICBT-I) may offer a scalable alternative to face-to-face insomnia treatment, requiring fewer specialized resources while reaching more people, without proportionally increasing clinician time and costs [53–56]. ICBT-I has general advantages that may be particularly preferable for autistic individuals: information can be processed at one’s own pace [57], it facilitates autonomy, and it’s predictable, structured format may reduce uncertainty and cognitive load [58, 59]. It may also alleviate some barriers and (sensory) difficulties experienced within face-to-face treatment [60–62]. Being able to receive treatment from home, and freely choose when to work on the intervention, is perceived as pleasant by autistic individuals [60]. Although not appropriate for everyone (e.g. those in high psychological distress or with intellectual disability) [63, 64], digital interventions have shown promise for supporting autistic individuals with social difficulties or other developmental targets [65, 66]. ICBT-I has been found to be feasible and effective in reducing insomnia in adolescents with autism [67]. To date, however, ICBT-I has not been studied in autistic adults.

In this randomized controlled trial, we adapted an existing, internet-delivered, CBT-I intervention (i-Sleep) in collaboration with autistic adults with insomnia. We evaluated the (long-term) effectiveness of “i-Sleep Autism” in guided self-help format compared to waitlist control with psychoeducation and sleep hygiene. Participants following the intervention were supported through asynchronous written feedback after each completed module. Our primary outcome was insomnia severity. Secondary outcomes included pre-sleep arousal, anxiety & depression, daily functioning and quality of life. At post-test, we expected a significant moderate effect on our primary outcome insomnia severity (*d* = 0.5) and positive effects on mental health: a decrease in anxiety, depression, and improvements in daily functioning and quality of life, as well as sustained effectiveness for 6 months.

## Materials and methods

### Study design

We conducted a two-arm, parallel, superiority randomized controlled trial to evaluate the effectiveness of a guided internet-delivered CBT-I intervention compared to a waitlist control group receiving online psychoeducation (PE) about sleep and information about sleep hygiene. Participants were randomized 1:1. After 6 weeks (end of intervention period), control participants were offered access to i-Sleep Autism. The study was approved by the Medical Ethical Committee of Amsterdam University Medical Centre (METC Amsterdam UMC) and registered in the Overview of Medical Research in the Netherlands (OMON) registry (NL-OMON56692). The protocol is available elsewhere [68].

### Eligibility criteria

Inclusion criteria for this study were: an ISI score of ≥10, as this cut-off has been deemed optimal for detecting insomnia cases [69], a formal autism diagnosis as established by an authorized professional, a minimum age of 18 years, and sufficient Dutch reading and writing abilities. Participants required access to a device (preferably laptop/tablet) with internet connectivity. Exclusion criteria included working in night shifts (defined as work between 2 a.m. and 6 a.m.), and current or planned pregnancy or breast feeding. Other co-occurring psychological or somatic conditions were not considered exclusion criteria, as these are highly prevalent among autistic individuals [70, 71], and CBT-I has demonstrated potential to improve co-occurring mental health symptoms [72, 73]. The use of (sleep) medication and cannabis was allowed and monitored during the study to enhance the external validity of the findings.

### Recruitment

Participants were recruited from the Netherlands Autism Register (NAR), a community-based longitudinal cohort of over 3000 autistic individuals in the Netherlands. The NAR collects annual online questionnaires to gain insight into the lives of autistic individuals in the Netherlands and inform autism knowledge, practice, and policy. Upon registration for the NAR, participants report details of their autism diagnosis, including when and where it was established, which professional(s) were involved, and the diagnostic criteria used (DSM-IV-TR or DSM-5).

Random batches of 50–150 adults were invited between May 2024 and August 2024 until the target sample of 160 participants was reached; in total, 1487 autistic adults were invited (Figure 1). Invitation emails included study information and a link to digital informed consent (Qualtrics). After completing this form, potential participants proceeded to an online screener. Eligible participants were automatically directed to the full baseline assessment. Ineligible participants were informed accordingly and provided with alternative resources for their sleep problems.

**Figure 1:**
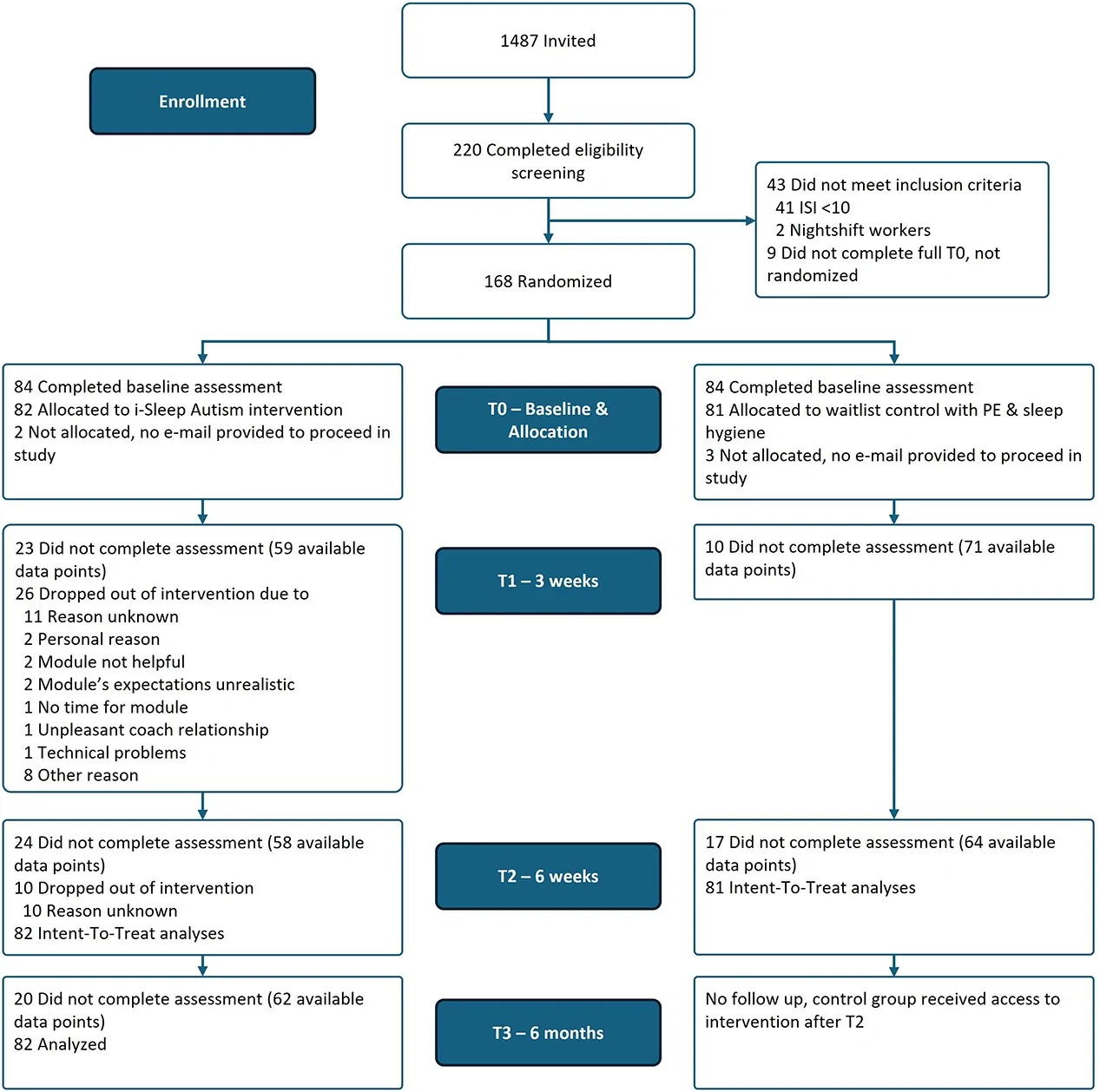
CONSORT flowchart diagram. *Note*. T1 intervention dropouts completed no more than two of five modules, while T2 dropouts completed no more than three. Participants could report multiple reasons for discontinuing the intervention.

### Randomization, blinding, and treatment allocation

Immediately after finishing the baseline assessment, a built-in Qualtrics randomizer assigned participants to one of the two conditions. Randomization was implemented using the Qualtrics “Evenly present elements” function, which dynamically adjusts assignment to either condition based on current group sizes to minimize imbalance in group size. No stratification was applied. Next, after filling out their e-mail address and choosing a preferred (anonymous) username for the intervention platform, participants were blindly allocated to the assigned condition by use of automated e-mailing schemes in Qualtrics. Thus, randomization and allocation procedures were fully blinded and automated. Due to the nature of the intervention, blinding of participants and coaches was not possible. The researcher responsible for the analysis (KS) was blinded for the study condition of participants.

### Procedures

Participant flow is illustrated in Figure 1. Within 24 hours after T0, participants randomized to the intervention group received instructions on how to activate their personal account at Minddistrict, an e-health platform used by health care organizations in the Netherlands to deliver digital therapy to their clients (https://www.minddistrict.com/). The intervention group received the guided i-Sleep Autism intervention, while the control group received online psychoeducation and sleep hygiene.

At 3 weeks (T1), both groups completed a brief questionnaire assessing the primary outcome and adverse events. At post-test (T2; 6 weeks), all baseline outcomes and adverse events were reassessed, with additional questions on intervention use and evaluation for the intervention group. After T2, the control group was offered i-Sleep Autism. At 6 months post-baseline (T3), follow-up assessments on all outcomes were completed by the intervention group only, as participants in the comparison group were offered access to the intervention after the post-treatment assessment (T2). Consequently, maintaining a non-intervention comparison group at six-month follow-up was not possible.

### Intervention

#### Intervention condition

**Intervention set up.** i-Sleep Autism is a guided, digital self-help intervention based on CBT-I. The intervention has 5 modules and addresses: (1) psychoeducation on normal sleep, sleep disorders, and sleep hygiene (factors and behaviors influencing sleep), (2) sleep scheduling (using fixed bedtimes) and restriction (limiting time in bed to increase sleep pressure) and stimulus control (strengthening the bed's association with sleep), (3) techniques to manage rumination and promote relaxation (progressive muscle relaxation, breathing exercises, meditation), (4) cognitive restructuring (changing common maladaptive thoughts about sleep), and (5) summary and approaches for relapse prevention.

Participants were advised to complete one module per week, resulting in a 5-week intervention. Each module took approximately 30–60 minutes and encompassed informative texts, audiovisual components (e.g. case examples, summary videos), and exercises. Participants were encouraged to integrate exercises into their daily routines. A brief daily online sleep diary accompanied the intervention. The program was accessed via the Minddistrict web platform, with sleep diaries completed through the Minddistrict smartphone application.

**Guidance by online coaches.** Participants were guided by online coaches (psychology students) who supported goal setting, motivation, and adherence through asynchronous written feedback using Motivational Interviewing (MI) techniques. Coaches received approximately 15 hours of training supervised by a licensed psychologist, including studying a coaching protocol, completing a CBT-I e-learning (LeerSlapen, Kempenhaeghe) [74], and attending a live one-day training with lectures on autism (characteristics, challenges, strengths, communication styles, neurodiversity affirming/inclusive language), CBT-I, and online coaching through written feedback. Two members of the co-creation panel joined the training to share their experiences, thoughts, and preferences.

Coaches provided the written feedback via the Minddistrict platform after each completed module (within three working days) and received bi-weekly supervision. The coaching period could be extended to 8 weeks if needed, and reminder messages were sent after 2 weeks of inactivity. Coaches reported adverse events, including suicidality, to the research team, with a licensed psychologist available for consultation and referral if required.

**Adaptation process.** i-Sleep has demonstrated effectiveness across various populations suffering from insomnia [75–78]. In collaboration with autistic adults experiencing insomnia and healthcare professionals, i-Sleep was adapted for people with autism while preserving the core components of CBT-I. A summary of overall adaptations and adaptations per module can be found in Supplementary Materials (Table S2).

The intervention was adapted in multiple iterative cycles of feedback. First, we conducted in-depth interviews with three healthcare professionals with experience with adults with autism and insomnia to identify important areas for adaptation. We then set up a panel of seven autistic individuals with insomnia (six females, one male, aged 24–57, varying educational backgrounds). For the panel, we scheduled an information session to explain the adaptation goals and planning, and to foster a safe, familiar environment for feedback. Panel members were asked to give feedback on content, language, usability, relevance, and acceptability for each module and overall, and to think of possible autism-friendly solutions. The feedback forms were structured in layout but highly open-ended to allow participants to raise issues freely (Supplementary Files “Feedback forms”). Written feedback was reviewed and synthesized by the research team and then discussed with the panel in two online focus groups. Based on this first round of feedback, the intervention was adapted and reviewed again by the panel through a second round of written feedback and one additional focus group. At this stage, feedback was minimal, so a final version of the intervention was created. Panel members could review this version and raise objections, but all participants were satisfied with this final version.

Adaptations were implemented when there was broad agreement among panel members and when proposed changes were compatible with the core principles of CBT-I. When panel members expressed conflicting views, decisions were guided by scientific literature and expert input. The adaptation process was pragmatic and informed by co-creation principles but was not based on a specific intervention adaptation framework. The following adaptations were made: the text was better matched to the situation of autistic individuals (e.g. psychoeducation on insomnia in autism specifically); extra emphasis was put on elements that were deemed important (e.g. light exposure, day time relaxation, bed time rituals); instructions were clarified and made informative rather than directive; more concrete examples were provided to provide better guidance with exercises; and case examples were replaced with three individuals with autism and insomnia sharing their experiences and practical tips (Table S2).

#### Control condition

Control participants received a digital folder with online psychoeducation and sleep hygiene, based on the first module of i-Sleep Autism (see “Intervention set up”). The information was therefore also adapted to autism, but did not include any exercises, videos or interactive components. Six weeks after their registration, after the T2 assessment, control participants gained access to the full i-Sleep Autism intervention and an online coach, if desired.

### Measures

#### Primary outcome

**Insomnia severity.** The Insomnia Severity Index (ISI) was used to measure the primary outcome, insomnia severity [79]. This scale consists of 7 items on a 5-point Likert scale ranging from 0 to 4, resulting in total scores between 0 and 28. Higher scores indicate greater insomnia severity. Scores are categorized as follows: 0–7 (no clinically significant insomnia), 8–14 (subthreshold insomnia), 15-21 (moderate clinical insomnia), and 22–28 (severe clinical insomnia). The ISI is validated for online applications [80], and showed excellent internal consistency (Cronbach’s α of .90–.91) [69]. The ISI has not been specifically validated for autistic adults, but has been used previously in autism research, e.g. by Hohn, de Veld, Mataw, van Someren and Begeer [23]. A 6-point reduction is considered a clinically meaningful improvement [81]. Cronbach’s alpha was .74 across our assessments.

#### Secondary outcomes

**Pre-sleep arousal.** The Pre-Sleep Arousal Scale (PSAS) was used to assess symptoms of cognitive and somatic arousal experienced at bedtime [82]. The 16 items are scored on a 5-point Likert scale ranging from 1 to 5, resulting in total scores between 16 and 80, with higher scores reflecting more arousal. The first 8 items measure somatic pre-sleep arousal (PSAS-somatic, ranging from 8 to 40), and the last 8 items measure cognitive pre-sleep arousal (PSAS-cognitive, ranging from 8 to 40). Scores are considered clinically relevant when they are ≥14 (somatic) and ≥20 (cognitive) [82]. The PSAS has previously shown good internal reliability in an autistic sample for the cognitive (Cronbach’s α = .89) and somatic (α = .81) subscales [83]. Across our assessments, Cronbach’s alpha was .83 for somatic and .88 for cognitive pre-sleep arousal.

**Anxiety and depression.** The Hospital Anxiety and Depression Scale (HADS) was used to assess anxiety and depression [84]. Seven items are dedicated to both anxiety and depression subscales, comprising a total of 14 items, scored on a 4-point Likert scale (0–3). Subscale total scores range from 0 to 21, with higher scores indicating greater symptom severity. Scores are categorized as follows: 0–7 (Normal), 8–10 (Mild), 11–15 (Moderate), and 16–21 (Severe). The HADS is a reliable and valid tool for assessing psychological distress, also in autistic individuals, with excellent convergent validity, acceptable divergent validity, and adequate to good internal consistency (HADS-A α .82-.84; HADS-D α .60-.72) [85]. Cronbach’s alpha was .84 for anxiety and .85 for depression across our measurements.

**Work and social adjustment.** The Work and Social Adjustment Scale (WSAS) was used to assess the extent to which insomnia interfered with daily functioning. It consists of 5 items assessing functioning in work, social and private leisure activities, home management, and the ability to form and maintain close relationships [86]. Each of the five items is rated on a 9-point Likert scale ranging from 0 to 8. Total scores range from 0 to 40, with higher scores indicating greater impairment. The WSAS has good internal consistency (Cronbach’s alpha of 0.70–0.94), and is sensitive to treatment effects [86, 87]. Cronbach’s alpha was .87 in this study.

**Quality of life.** The Mental Health Quality of Life Questionnaire (MHQoL) was used to assess quality of life [88]. The seven items encompass self-image, independence, mood, relationships, daily activities, physical health, and future. They are scored on a 4-point Likert-scale (0–3). Total MHQoL scores range from 0 to 21, with higher scores indicating better quality of life. The MHQoL also includes a general visual analogue scale (VAS) for overall psychological wellbeing. The VAS ranges from 0 to 10, where higher scores represent higher psychological wellbeing. The MHQoL has demonstrated strong internal consistency (Cronbach’s α = .85) and was designed to identify the effects of mental health interventions [88]. The average Cronbach’s alpha was .77 in this study.

#### Intervention evaluation

In addition to the standardized questionnaires assessing our participants’ satisfaction and alliance with the intervention and the coaches (described below), we asked whether they preferred the online format over face-to-face or vice versa. We also asked what exercises they put most effort into.

**Client satisfaction.** The Client Satisfaction Questionnaire adapted to Internet-based interventions (CSQ-I) assessed partici-pant’s satisfaction with the intervention [89]. Although the study protocol initially specified the use of the CSQ-8 [68], the CSQ-I was selected instead, as it was considered more appropriate for evaluating satisfaction with our online intervention. The eight items are scored on a 4-point Likert-scale (1–4). Total scores range from 8 to 32, with higher scores denoting higher satisfaction. The CSQ-I has good internal consistency, factorial structure, and construct validity as an instrument (McDonald omega of .93-.95) [89]. Cronbach’s alpha was .88 in our study.

**Working alliance.** The Working Alliance Inventory-Short Revised (WAI-SR) assessed therapeutic alliance between participants and their coaches [90]. The scale includes three key aspects of therapeutic alliance: task agreement, goal agreement, and development of an effective bond between client and therapist. The 12 items are scored on a 5-point Likert scale (1–5). Total scores range from 12 to 60, subdomain total scores range from 4 to 20, with higher scores indicating higher therapeutic alliance. The WAI-SR exhibits good reliability (alpha > .8) and convergent validity (r > .64) [91]. Internal reliability was excellent in this study (Cronbach’s alpha = .97).

**Working—and technical alliance.** The Working Alliance and Technical Alliance Short Form (WAI-TECH-SF), a modified version of the WAI-SF, assessed therapeutic alliance between clients and our online intervention [92]. The 12 items are scored on a seven-point Likert scale, ranging from 1 to 7. Total scores range from 12 to 84, subscales range from 4 to 28. The scale maintains the same structural dimensions as the WAI-SR (therapeutic goals, tasks, and bonds). The scale has excellent reliability (Cronbach’s α = .97) [92], similarly, Cronbach’s alpha was .98 in this study.

**Adverse events.** Adverse events were assessed at T1 and T2 in both groups, through the following questions: "Have you experienced anything unpleasant since the previous questionnaire 3 weeks ago as a result of your sleep problem or as a result of your participation in the study? For example: suicidality, fall accidents, traffic accidents or other incidents due to fatigue and drowsiness?" (No/Yes, namely..) and “Was medical or psychological assistance needed in relation to this incident?” (No/Yes, namely..).

#### Descriptive variables

Demographic variables, including age, sex, education level, relationship status, and living situation, and co-occurring conditions were obtained from the NAR database and the annual survey conducted prior to data collection for this study (spring 2024). All NAR participants provided informed consent allowing their data to be used for research purposes.

Given the relevance of these variables to sleep outcomes, baseline data on alcohol and drug (ab)use were collected using the Alcohol Use Disorders Identification Test (AUDIT-10) and the Drug Abuse Screening Test (DAST) [93, 94]. Additionally, information on the type and frequency of healthcare use and sleep medication usage in the past 2 weeks (yes/no) was gathered for both study arms throughout the trial.

### Power and sample size calculation

Compared to the large effect sizes observed in meta-analyses establishing the effect sizes of CBT-I interventions [38, 95], we powered conservatively for a medium effect size of d = 0.50 on the primary outcome insomnia severity at the post-test measurement. To ensure 80% statistical power with a 2-sided α-value of 0.05, we estimated that a total number of 128 participants (64 per group) was required. Anticipating a 20% risk of dropout [38], we aimed to recruit 160 participants (80 per group).

### Statistical analyses

All analyses were performed using SPSS version 27 and R version 4.4.2, with a two-tailed significance level set at α = 0.05. To account for the hierarchical structure of the data (repeated measures nested within participants), Linear Mixed Models (LMMs) were applied throughout.

#### Model specification

For each outcome, a null model with a random intercept for subject was fitted to assess clustering. For insomnia severity, the ICC was 0.54, indicating substantial between-subject variance; the resulting design effect was 1.84, yielding an effective sample size of n = 226.

Random intercept and slope models and covariance structures were compared using AIC and BIC. An AR(1) heterogeneous structure provided the best fit for insomnia severity, while a standard AR(1) structure was preferred for secondary outcomes. Due to convergence issues, final models included a random intercept for subjects only.

#### Primary and secondary outcomes

We tested our first hypothesis—whether the intervention led to a greater reduction in insomnia severity (ISI) compared to the control group between baseline and post-test (T0–T2)—using intention-to-treat (ITT) analyses. All participants were analyzed as originally assigned, regardless of adherence. To assess robustness, per-protocol (PP) analyses were also conducted including only intervention participants who completed modules 1 through 4 (considered the core elements, as module 5 provides a summary and focuses on relapse prevention).

In the final two-level LMM (time nested within participants), fixed effects included group (0 = intervention, 1 = control), time (1 = post-test, 2 = mid-intervention, 3 = baseline), and their interaction (group × time). A random intercept for subjects was included. Models were estimated using Restricted Maximum Likelihood (REML).

Baseline-adjusted Cohen’s *d* effect sizes were calculated and interpreted as small (*d* < 0.20), medium (*d* = 0.50), and large (*d* > 0.80). Chi-square tests compared remission rates (ISI ≤ 9 vs. ≥ 10) and clinically meaningful change (improvement: ≥6-point decrease; deterioration: ≥6-point increase; no change: −5 to +5). Odds ratios were computed for improvement and remission.

The same LMM approach was used to test effects on secondary outcomes: pre-sleep arousal (PSAS), anxiety/depression symptoms (HADS), work and social adjustment (WSAS), and quality of life (MHQoL). Descriptive statistics of the client satisfaction with the intervention (CSQ-I), working alliance with the coach (WAI-SR) and the intervention (WAI-TECH-SF) were obtained to evaluate the intervention.

#### Moderation and predictors

Potential moderators of treatment effect on insomnia severity were explored using interaction terms in the LMM. Moderator variables included baseline ISI, insomnia duration (years), age, sex, and presence of a co-occurring diagnosis. Continuous moderators were grand-mean centered before inclusion. Significant moderators were investigated using simple slope analysis. Additionally, we assessed the bivariate correlation between therapeutic alliance (WAI-SR) and ISI change scores.

#### Long-term follow-up

To explore the long-term robustness of the results, we conducted within-group Repeated Measures ANOVAs for the intervention group only, contrasting post-test (T2) and six-month follow-up (T3).

## Results

### Sample descriptives

A total of 163 autistic adults participated in this study. The average age was 48.5 years, 59.5% of participants was female, 60.7% highly educated and 55.4% self-reported one or more co-occurring conditions next to autism and insomnia, e.g. ADHD and PTSD (Table 1). Sleep medication use in the past 2 weeks at post-test was similar to baseline (Table S1). There were no significant baseline differences between the groups on any of the outcome variables.

**Table 1:** Demographics and clinical characteristics at baseline

	Control	Intervention	Total
Characteristic	*n* = 81	*n* = 82	*n* = 163
Age, mean (SD), years	49.38 (14.20)	47.59 (13.32)	48.46 (13.74)
Biological sex, female n (%)	46 (56.8%)	51 (62.2%)	97 (59.5%)
Autism Quotient, mean (SD)	85.36 (12.00)	85.35 (10.50)	85.35 (11.21)
Paid job, n (%)	35 (43.2%)	37 (45.1%)	72 (44.2%)
Highest obtained education, n (%)			
Low	12 (14.8%)	4 (4.9%)	16 (9.8%)
Medium	19 (23.5%)	22 (26.8%)	41 (25.2%)
High	45 (55.6%)	54 (65.9%)	99 (60.7%)
Intellectual ability, n (%)			
IQ < 86	7 (8.6%)	6 (7.3%)	13 (7.1%)
IQ 86-115	15 (18.5%)	19 (23.2%)	34 (20.9%)
IQ > 115	53 (65.4%)	54 (65.9%)	107 (65.6%)
Living situation, n (%)			
Living alone	28 (34.6%)	43 (52.4%)	71 (43.6%)
Living with partner and/or children	42 (51.9%)	33 (40.2%)	75 (46.0%)
Living with parents/caretakers	4 (4.9%)	3 (3.7%)	7 (4.3%)
Other	1 (1.2%)	2 (2.4%)	3 (1.8%)
Co-occurring condition, n (%)	45 (55.6%)	42 (51.2%)	87 (53.4%)
Depressive disorder	21 (25.9%)	18 (22.0%)	39 (23.9%)
AD(H)D	13 (16.0%)	14 (17.1%)	27 (16.6%)
Anxiety disorder	13 (16.0%)	13 (15.9%)	26 (16.0%)
PTSD or trauma	10 (12.3%)	15 (18.3%)	25 (15.3%)
Burnout	7 (8.6%)	6 (7.3%)	13 (8.0%)
Personality disorder	6 (7.4%)	5 (6.1%)	11 (6.7%)
Visual impairment	4 (4.9%)	6 (7.3%)	10 (6.1%)
OCD and related disorders	5 (6.2%)	4 (4.9%)	9 (5.5%)
Learning disability	3 (3.7%)	6 (7.3%)	9 (5.5%)
Other^a^	16 (19.8%)	21 (25.6%)	37 (22.7%)
Sleep medication (past 2 weeks), n (%)	32 (39.5%)	27 (32.9%)	59 (36.2%)
Benzodiazepines & Z-drugs	17 (20.2%)	14 (17.1%)	31 (19.0%)
Melatonin receptor agonist	10 (12.3%)	4 (4.9%)	14 (8.6%)
Antipsychotic	7 (8.6%)	4 (4.9%)	11 (6.7%)
Antidepressant	5 (6.2%)	4 (4.9%)	9 (5.5%)
Alternative remedies	5 (6.2%)	2 (2.4%)	7 (4.3%)
Antihistamine	1 (1.2%)	2 (2.4%)	3 (1.8%)
Cannabis/CBD product	2 (2.5%)	1 (1.2%)	3 (1.8%)
Other^b^	2 (2.5%)	2 (2.4%)	4 (2.5%)
Duration of sleep medication use (past 2 weeks), mean (SD), days	10.16 (3.84)	9.17 (5.40)	9.71 (4.60)
AUDIT-10, mean (SD)	2.48 (3.46)	2.79 (4.08)	2.69 (3.77)
DAST-10, mean (SD)	0.19 (0.84)	0.28 (1.08)	0.24 (0.97)
Insomnia duration, mean (SD), years	18.69 (15.88)	18.80 (17.89)	18.74 (16.87)

*Note.* Control: Waitlist control with psychoeducation and sleep hygiene; Intervention: i-Sleep Autism; Alcohol Use Disorders Identification Test (AUDIT-10); Drug Abuse Screening Test (DAST-10). Intellectual ability was self-reported based on test results or estimated guess. Participants could report more than one sleep medication and co-occurring condition, so category totals may exceed the number of participants reporting any medication or condition.

a Other reported co-occurring conditions were combined into one category, conditions were reported 1-6 times. Examples include auditory impairment, eating disorders, and epilepsy.

b Other medication used for sleep: methylphenidate, acetaminophen, and topiramate.

### Intervention adherence

Of all intervention participants, 50% completed the full intervention (5 modules), 81.7% completed one or more, 68.3% completed two or more, 58.5% completed three or more, 56.1% completed 4 modules (PP analyses), and 18.3% never started (Table S3, Supplementary Materials). Post-hoc, exploratory comparisons (t-tests and chi-square tests) of age, sex, co-occurring conditions, and baseline ISI scores between intervention completers and non-completers showed no significant differences. Adherence to the recommended schedule was generally good for participants and coaches. Those participants who completed all five modules did so in 40.8 days on average, approximately six days longer than the intended 5-week program. Coaches provided feedback within 3.7 working days on average, slightly more than the intended three working days.

### Intervention effects on primary and secondary outcomes from baseline to post-test

Compared to control, the i-Sleep Autism intervention reduced our primary outcome insomnia severity at 3 weeks with medium effect size (T1; *d* = 0.49; SE of *d* = 0.18; 95% CI of *d* [0.14, 0.84]; B = -1.33; 95% CI of B [-2.46, -0.21]) and large effect at post-test (T2; *d* = 1.02; SE of *d* = 0.18; 95% CI of *d* [0.65, 1.40]; B = -3.97; 95% CI of B [-5.52, -2.42]) (Tables 2 and 3; Figure 2). For secondary outcomes, the intervention group showed greater reductions in total, cognitive, and somatic pre-sleep arousal and higher quality of life at post-test compared to controls (Tables 2 and 3). No significant effects were found for anxiety, depression, or daily functioning. All these analyses were performed on ITT principle.

**Table 2:** Unadjusted mean scores for the post-test and 6 month follow up, baseline-adjusted effect sizes

	Baseline, M(SD)		6 weeks, M(SD)		Cohen’s *d*		6 months, M(SD)	
	Control (*n* = 81)	Intervention (*n* = 82)	Control (*n* = 64)	Intervention (*n* = 58)	*d* (SE)	95%CI	Control	Intervention (*n* = 59)
Insomnia severity	16.21 (3.31)	15.54 (4.12)	14.95 (4.32)	10.05 (5.35)	**1.02 (0.19)**	**0.65; 1.40**	−	10.37 (5.10)
Pre-Sleep Arousal	44.30 (11.14)	42.51 (11.81)	40.91 (11.08)	32.38 (10.80)	**0.73 (0.19)**	**0.37; 1.10**	−	34.56 (9.50)
Cognitive	26.56 (6.51)	25.32 (6.80)	26.56 (6.51)	18.86 (6.47)	**0.78 (0.19)**	**0.42; 1.15**		20.31 (6.21)
Somatic	17.74 (5.94)	17.20 (6.82)	17.74 (5.94)	13.52 (5.45)	**0.50 (0.18)**	**0.13; 0.86**		14.25 (4.54)
Anxiety	10.81 (4.06)	9.66 (4.63)	10.16 (3.23)	7.83 (2.99)	0.23 (0.18)	−0.12; 0.59	−	7.95 (4.38)
Depression	9.74 (4.55)	8.46 (5.16)	9.06 (4.77)	7.21 (5.14)	0.29 (0.18)	−0.06; 0.65		6.98 (5.12)
Daily functioning	18.22 (7.85)	16.60 (8.58)	15.56 (8.70)	14.59 (10.04)	0.17 (0.18)	−0.18; 0.53	−	12.47 (9.79)
Quality of life	12.67 (3.01)	12.94 (3.10)	11.52 (3.45)	12.97 (3.49)	**0.39 (0.18)**	**0.03; 0.75**	−	12.58 (3.43)

*Note.* Insomnia Severity Index (ISI); Pre-Sleep Arousal Scale (PSAS); Hospital Anxiety and Depression Scale (HADS); Work and Social Adjustment Scale (WSAS); Mental Health Quality of Life (MHQoL). Significant effects are indicated in bold. Cohen’s *d* effect sizes were adjusted for baseline scores.

**Table 3:** Estimated effects from linear mixed models for primary and secondary outcomes based on ITT principle

	Intercept	T1 (3 weeks)	T2 (6 weeks)	Group	Group ^*^ T1	Group ^*^ T2
Insomnia severity						
B (SE)	16.21 (0.42)	−1.87 (0.39)	−1.34 (0.54)	−0.67 (0.59)	**−1.33 (0.57)**	**−3.97 (0.79)**
95% CI	15.38; 17.04	−2.64; −1.11	−2.41; −0.27	−1.84; 0.50	**−2.46; −0.21**	**−5.52; −2.42**
PSAS (Total)						
B (SE)	44.30 (1.26)	−	−3.72 (1.08)	−1.78 (1.78)	−	**-5.30 (1.57)**
95% CI	41.80; 46.80	−	−5.86; −1.57	−5.30; 1.73	−	**−8.40; −2.19**
PSAS (Cognitive)						
B (SE)	26.56 (0.75)	−	−2.33 (0.63)	−1.24 (1.05)	−	**-3.44 (0.91)**
95% CI	25.10; 28.02		−3.57; −1.09	−3.31; 0.84		**−5.23; −1.65**
PSAS (Somatic)						
B (SE)	17.74 (0.68)	−	−1.39 (0.63)	−0.55 (0.96)	−	**−1.87 (0.92)**
95% CI	16.40; 19.08	−	−2.65; −0.14	−2.44; 1.35	−	**−3.68; −0.06**
Anxiety						
B (SE)	10.16 (0.38)	−	−1.06 (0.34)	−0.87 (0.34)	−	−0.25 (0.49)
95% CI	9.41; 10.91	−	−1.74; −0.38	−1.93; 0.19	−	−1.22; 0.73
Depression						
B (SE)	9.74 (0.55)	−	−0.51 (0.40)	−1.28 (0.77)	−	−0.69 (0.58)
95% CI	8.66; 10.82	−	−1.74; −0.38	−2.81; 0.25	−	−1.84; 0.46
Daily Functioning						
B (SE)	18.22 (0.97)	−	−2.95 (0.88)	−1.63 (1.37)	−	−0.94 (1.22)
95% CI	16.31; 20.13	−	−4.66; −1.31	4.32; 1.07	−	−3.36; 1.48
Quality of Life						
B (SE)	12.67 (0.36)	−	−1.18 (0.33)	0.27 (0.51)	−	**1.00 (0.48)**
95% CI	11.96; 13.38	−	−1.83; −0.52	−0.73; 1.27	−	**0.06; 1.95**

*Note.* ITT = Intent-To-Treat; B = unstandardized B-coefficient; SE = Standard Error. Significant intervention effects in bold. The baseline (T0) and the control group were set as reference. The estimated parameters represent: Intercept: mean score of the control group at T0; Time 1: control group’s estimated change in outcome variable between T0 and 3 weeks (T1); Time 2: control group’s estimated change in outcome variable between T0 and 6 weeks (T2); Group × time interaction effects indicate the additional impact of i-Sleep Autism compared to the control group at T1 and T2. Primary outcome: Insomnia Severity Index (ISI), assessed at T0, T1, and T2. Secondary outcomes: assessed at T0 and T2—Pre-Sleep Arousal Scale (PSAS; cognitive and somatic subscales), Hospital Anxiety and Depression Scale (HADS; anxiety and depression subscales), Work and Social Adjustment Scale (WSAS), and Mental Health Quality of Life (MHQoL). Significant interaction effects are shown in bold. All parameter estimates are model-based adjusted estimates derived from LMMs

**Figure 2:**
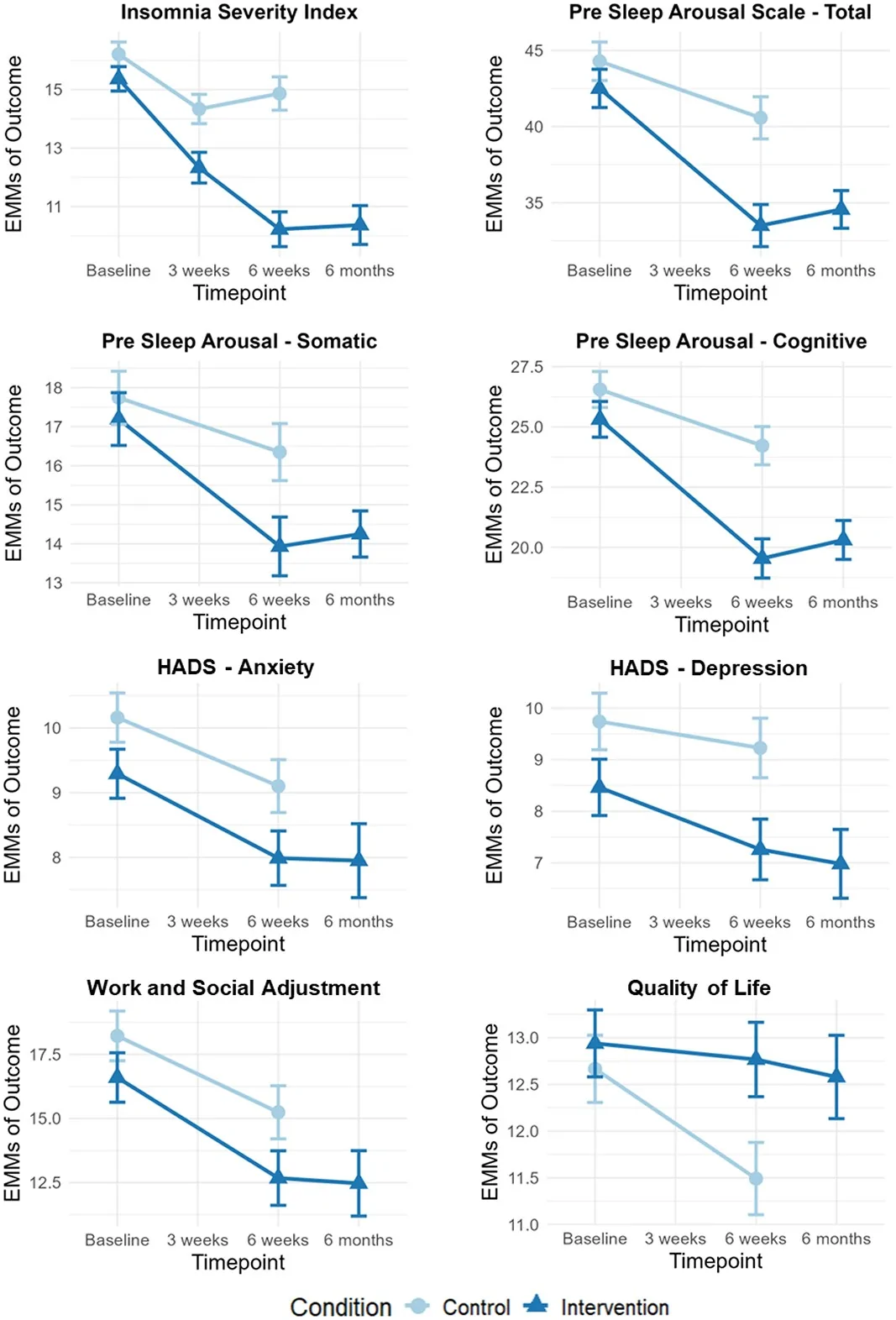
Interaction effects of all outcomes (primary and secondary). *Note*. Estimated marginal means (EMMs). Final assessment (6-months) is an uncorrected mean. Higher scores indicate greater symptom severity or impairment for the ISI, PSAS, HADS-A/D, and WSAS, and better quality of life for the MHQoL.

#### Sensitivity analyses

Per-Protocol analyses comparing those who completed four modules (*n* = 46) with controls showed larger effects on insomnia severity at T1 (*d* = 0.5, 95% CI [0.14, 0.86]) and T2 (*d* = 1.39, 95% CI [0.99, 1.78]), PSAS total (*d* = 1.1, 95% CI [0.72, 1.48]), PSAS cognitive (*d* = 1.05, 95% CI [0.67, 1.43]), PSAS somatic (*d* = 0.86, 95% CI [0.48, 1.23]), and quality of life (*d* = 0.56, 95% CI [0.19, 0.92]). Depression was significantly reduced with medium effect size in PP but not ITT analyses (*d* = 0.52, 95% CI [0.16, 0.89]). Additional post hoc sensitivity analyses showed that neither changes in sleep medication use from baseline to post-test nor the frequency of sleep medication use at post-test were significant predictors of post-test ISI scores, and inclusion of these variables did not alter the main treatment effect.

#### Clinically meaningful change and remission for insomnia severity at post-test

No participants showed clinical deterioration (Table 4). There was a significant difference between the intervention and control group: intervention participants had 7.0 times higher odds of clinical improvement (SE = 0.45, 95% CI [2.9, 16.8]) and 4.5 times higher odds of remission (SE = 0.44, 95% CI [1.94, 10.66]). Confidence intervals for these findings are wide, and only complete cases were analyzed.

**Table 4:** Clinically meaningful change and remission rates

	WL Control with PE and SH (*n* = 81)		Guided ICBT-I (*n* = 82)		χ^2^(1, n = 122)	*p*	Odds Ratios (95% CI)
Insomnia Severity (ISI)	*n*	(%)	*n*	(%)			
Unchanged	55	67.9	27	32.9	21.42	<.001	
Clinically improved	9	11.1	31	37.9			7.02 [2.93, 16.81]
Unknown	17	21.0	24	29.3			
Total							
No remission	55	67.9	29	35.4	18.32	<.001	
Remission	9	11.1	29	35.4			4.54 [1.94, 10.66]
Unknown	17	21.0	24	29.3			
Total							

*Note.* Missing post-test data were excluded from these analyses (complete-case analysis), percentages displayed in this table were calculated respective to the full group sizes post randomization (ITT). Clinically meaningful change was defined as a ≥ 6-point change on the Insomnia Severity Index (ISI): improvement (≥6-point decrease), deterioration (≥6-point increase), and unchanged (<6-point change). Remission was defined as ISI ≤ 9 at post-test.

#### Moderators and predictors of the post-test intervention effect

Exploratory analyses showed that only age moderated the intervention effect at 6 weeks (B = 0.11, SE = 0.05, *p* = .014), with stronger effects for younger than older participants (Figure S1). The intervention effect was stronger for younger participants (age -1SD: difference = 6.57, 95% CI [4.50, 8.65], *p* < .001) compared to older participants (age + 1SD: difference = 2.70, 95% CI [0.69, 4.71], *p* = .009) (Figure S1). Stronger therapeutic alliance was associated with greater reductions in insomnia severity (*r* = −.338, *p*=.003; Figure S2).

#### Adverse events

Adverse events, defined as negative events related to sleep problems or study participation, were assessed mid-intervention (3 weeks) and at post-test (6 weeks). Reported events included falls, minor injuries, and social conflicts due to tiredness. At 3 weeks, 8 participants in the control group (9.9%) and 5 in the intervention group (6.1%) reported an adverse event. At 6 weeks, this was 5 (6.2%) and 6 (7.3%), respectively. In the intervention group, one participant required medical support for a dislocated ankle, and one participant required psychological support due to autistic meltdowns at both assessments.

#### Long-term effects within the intervention group

Within-group intervention effects were largely maintained at six-month follow-up, with moderate increases only in total and cognitive pre-sleep arousal, reflecting more pre-sleep arousal with moderate effect sizes (Figure 2, Table 5).

**Table 5:** Within-group repeated measures ANOVA effects for the intervention group at 6 months versus baseline and post-test

Predictor	Contrast	df	F	Sig.	*p* ^2^
Insomnia severity	T2 – T3	1,50	2.248	.140	.043
Pre-sleep arousal—total	T2 – T3	1,52	7.401	**.009**	.125
Pre-sleep arousal—cognitive	T2 – T3	1,52	6.293	**.015**	.108
Pre-sleep arousal—somatic	T2 – T3	1,52	2.369	.130	.044
Anxiety and depression—Total	T2 – T3	1,52	0.174	.679	.003
Anxiety	T2 – T3	1,52	0.097	.757	.002
Depression	T2 – T3	1,52	0.093	.761	.002
Work and social adjustment	T2 – T3	1,52	1.990	.164	.037
Quality of Life	T2 – T3	1,52	2.156	.148	.040

Note. T0: Baseline; T2: Post-test, 6 weeks; T3: Follow up, 6 months. Significant within-group changes from T2 to T3 are indicated in bold.

### Intervention evaluation

Overall satisfaction with the intervention was moderate to high (M = 21.12, range 9–30; Table S4). Therapeutic alliance with the coach was high (M = 39.1, range 12–60) and comparable across Goal, Task, and Bond subscales. Perceived alliance with the online intervention showed substantial variability (M = 49.68, SD = 20.72).

Most participants (70.7%) preferred the online format over face-to-face, mainly due to flexibility and the ability to work at their own pace (Table S5). Preference for face-to-face delivery was primarily related to missing direct interaction and personal contact with the therapist (Table S6).

Participants reported investing the most effort in the following intervention components (from most to least effort): evaluating and altering sleep hygiene, creating a bedtime routine, keeping fixed bedtimes, and consciously dealing with light exposure. The least effort was reportedly put into cognitive exercises aimed at reducing worrying and rumination, and altering dysfunctional thoughts about sleep.

## Discussion

In this parallel, superiority randomized controlled trial, i-Sleep Autism significantly reduced insomnia severity compared to a waitlist control receiving online psychoeducation and sleep hygiene with a large effect size. Nearly 40% of participants achieved clinically meaningful improvement, with sevenfold higher odds of improvement than controls. The intervention also reduced pre-sleep arousal with medium to large effect and improved quality of life with small to medium effect, but showed no significant effects on daily functioning, anxiety, or depression (except in per-protocol analyses for depression, where a medium effect was observed). Effects were stronger among younger participants and those reporting greater therapeutic alliance, and were largely maintained at 6-month follow-up. Overall, the intervention was positively evaluated.

To our knowledge, this is the first ICBT-I intervention specifically tested in autistic adults [43]. The effectiveness of our intervention on insomnia severity (primary outcome) is comparable to effects observed of guided ICBT-I in the general population (umbrella review: d = 1.05 at post-test) [39]. It must be noted, however, that waitlist control designs can inflate effect sizes [96], and the observed effects may partially reflect non-CBT-I-specific factors such as guidance and structured engagement. Future studies could compare guided ICBT-I with other active comparators such as in-person (group) CBT-I to evaluate its relative effectiveness in autism. Rates for clinically meaningful reduction of insomnia are also comparable (37.9% clinically meaningful change; 35.4% remission) to effects of guided ICBT-I in the general population [39], although these percentages may decline when higher ISI cut-offs (e.g. ≥8 points) are applied. The average within-group reduction in insomnia severity was fully maintained at 6-month follow up, whereas previous studies report attenuation of treatment effects over time [37]. This suggests that, in accordance with our hypotheses, ICBT-I is effective in reducing insomnia severity in autistic individuals, also 6 months later.

Total, cognitive and somatic pre-sleep arousal significantly decreased at T2, which may have mediated the intervention’s effects on insomnia severity [97]. We only found significant moderate to large effects for depression and not anxiety in our Per-Protocol analyses, whilst recent meta-analyses of the effects of ICBT-I on anxiety and depression found small to moderate effects for these outcomes respectively [98, 99]. Functional impairment related to insomnia also did not improve significantly. This may reflect a true absence of effects on these outcomes in autistic adults, or a lack of power to detect the relatively small effects observed. For quality of life, CBT-I is generally found to have a moderate positive effect [100]. We primarily observed a deterioration in the control group, potentially reflecting either protective effects of the intervention or challenges faced on a waitlist or both [101].

Among participants who evaluated the intervention, overall satisfaction and therapeutic alliance with the coach were moderate to high, and most participants preferred the online format over face-to-face delivery because of its flexibility and self-paced nature, suggesting generally favorable acceptability, although the perspectives of dropouts are not integrated into this evaluation. Post-test dropout in the intervention group (29.3%) and full treatment adherence (completion of 5 modules) (50%) were both less favorable than rates reported for guided ICBT-I in patients with co-occurring conditions (18% study dropout; 69.2% full treatment adherence) [102] and in previous i-Sleep studies in other populations (68–85.7% adherence) [77, 78, 103], indicating lower feasibility within this population. Most intervention dropouts occurred early, with participants either not starting (18%) or discontinuing after modules 1 or 2 (21%). Non-initiation may have been related to recruitment during the summer holiday period. Reported reasons for later dropout included limited perceived benefit, high assignment burden, and frustration with platform or sleep diary usability, although many reasons remain unknown. These findings highlight the importance of thorough platform testing and user-friendly design, particularly for autistic adults. Planned platform improvements include enhancing accessibility through the implementation of a more natural text-to-speech function and improving the sleep diary through automatic calculation and clearer presentation of sleep parameters.

Although no participants reliably deteriorated, 32.9% showed no clinically meaningful improvement. While the digital format offers flexibility, its effectiveness depends on personal accountability, technological skills, and confidence [104]. Consistent with findings in the general population, effects were somewhat stronger among younger participants, possibly reflecting greater digital affinity [105, 106]. For some participants, support may have been insufficient or treatment demands too high. Sleep restriction was experienced as particularly challenging, and some exercises were interpreted very rigidly, potentially increasing stress. More interactive formats (e.g. video or face-to-face guidance) might allow greater flexibility in the interpretation and application of exercises. While findings of drop-out and non-response are common in internet-delivered interventions, certain autism-related characteristics (e.g. rigidness) may have contributed to engagement difficulties. Further analyses of non-response and dropout are needed to refine the intervention.

The intervention may also have been less suitable for individuals with high psychological distress or lower intellectual functioning [63, 64], given its cognitive and behavioral demands. Suitable alternatives should be explored for these groups. There are also indications that circadian sleep–wake disorders are prevalent in autism [19, 107] next to having a sole insomnia disorder. Even though our study showed improved sleep, targeting both disorders at the same time might lead to even better outcomes. The transdiagnostic approach of Harvey (Transdiagnostic Intervention for Sleep and Circadian Dysfunction, TranS-C) is a promising candidate for that [108]. Adjunctive approaches such as chronotherapy or light therapy may also be beneficial in such cases [109]. Future studies need to examine if these approaches would be even more effective in this target group than treating insomnia alone.

Future research should disentangle which specific content and delivery components drive change in autistic people with insomnia and which require adaptation. Core behavioral elements such as sleep restriction and stimulus control may have contributed most to symptom improvement [110], despite being challenging for autistic individuals due to their impact on predictability and routines [51]. In contrast, sleep hygiene and psychoeducation were used most frequently by our sample but have relatively weak empirical support [36], and may even feel redundant or frustrating for individuals with long-standing insomnia, as reflected in qualitative feedback received through e-mail during and after the trial (e.g. “I’ve tried these tips for years”). Cognitive restructuring, although generally effective in targeting dysfunctional sleep beliefs [111], was applied least often in our sample, which may relate to its complexity in an online format and/or lower acceptability of cognitive restructuring among autistic individuals [112]. Future research should address these concerns, and alternatives such as ACT should be investigated for autistic individuals who do not respond well to cognitive restructuring [46].

Despite opportunities for refinement, the large effect size and the absence of deterioration seem to indicate that i-Sleep Autism can be offered safely in a broader non-clinical context (e.g. open online platform). Given the high prevalence of insomnia and sleeping problems in autistic individuals [4, 6–8], and the large impact on their lives [13, 14], this intervention could play an important role in improving these problems. The program could be implemented as stand-alone intervention, waiting-list care or as a component of blended care, although these latter applications require formal evaluation, including cost-effectiveness analyses.

Guided internet-based CBT-I may be a promising alternative to face-to-face CBT-I in view of the limited number of trained therapists [113] and the student coaches’ modest time investment per participant (<30 minutes per module) in this study. Further work should examine which coach characteristics (e.g. training in CBT-I, familiarity with autism, communication style) enhance outcomes, and to what extent these preferences from clients align with objective effectiveness, in order to balance personalization with scalability. Finally, insights from this trial may help refine CBT-I protocols for people with neurodevelopmental conditions and insomnia more broadly [43].

The strengths of our study lie in its robust design, large sample size, and our strong focus on collaboration with autistic individuals throughout the research process, including the tailoring of the i-Sleep Autism intervention and the interpretation and writing of this paper. As we aimed to establish the “real-world” effect of assigning the intervention, the study was pragmatic in nature, i.e. the eligibility criteria were broad, and participants were given substantial flexibility in when and how they engaged with the intervention, with minimal reminders, and coaches were not subject to strict protocol monitoring. However, the generalizability of our findings to the broader autistic group may still be limited, as our participants were mainly female, highly educated, and of Dutch nationality, and recruited through an active longitudinal cohort. It remains unclear how the intervention would be received in more socioeconomically and culturally diverse settings. Furthermore, not all included measures such as the ISI, WSAS and MHQoL have been validated specifically for autistic people, so they may lack sensitivity for this population. Other sleep disorders were not assessed in this sample, limiting our ability to determine whether comorbid sleep disorders such as sleep apnea were present and may have influenced insomnia severity and treatment response. Lastly, suicidality was not included as a study outcome, despite the known associations between insomnia, depression, and suicide risk [114], and the elevated prevalence of suicidal ideation and behavior among autistic adults [115]. Future research should examine whether improvements in insomnia are associated with reductions in suicidal ideation and behavior in this population.

## Conclusion

All in all, i-Sleep Autism appears to be acceptable, and to effectively and sustainably improve insomnia, considering the large effect size, more than one third experiencing clinically meaningful reduction of insomnia severity, and average appreciation. Given its scalability and relatively low resource requirements, guided ICBT-I represents a promising and accessible treatment option for autistic adults, a population with a high prevalence of insomnia. However, the relatively high study- and intervention drop-out rates suggest that the feasibility of the intervention should be enhanced. Future efforts should aim to support those that dropped out or did not benefit from the intervention.

## Supplementary Material

Supplementary_materials_zsag199

## Data Availability

The data collected in this study are available from the corresponding author upon reasonable request.

## Footnotes

1 In this research paper, identity-first and person-first language are used interchangeably, as studies report varying preferences among the autistic community [1].

## Clinical trial information

This study was registered at Overview of Medical Research in the Netherlands (OMON) under the title “Effectiveness of a guided e-health CBT-I based intervention for insomnia in autistic adults (i-Sleep Autism)”. URL: https://onderzoekmetmensen.nl/en/trial/56692. Registration number: NL-OMON56692.

## Trial status

The trial has been completed. Recruitment ran from May 2024 till August 2024. Data collection ran from May 2024 to February 2025.

## References

[1] Schuck RK, Chetcuti L, Dwyer P, et al. Preferences for identity-first and person-first language: a systematic review of research with autistic adults/adults with autism. *J Autism Dev Disord*. 2025. Advance online publication. 10.1007/s10803-025-07174-3 41389164

[2] Nogueira HA, de Castro CT, da Silva DCG, Pereira M. Melatonin for sleep disorders in people with autism: systematic review and meta-analysis. *Prog Neuropsychopharmacol Biol Psychiatry*. 2023;123:110695. 10.1016/j.pnpbp.2022.110695 36584862

[3] Ballester P, Richdale AL, Baker EK, Peiró AM. Sleep in autism: a biomolecular approach to aetiology and treatment. *Sleep Med Rev*. 2020;54:101357. 10.1016/j.smrv.2020.101357 32759030

[4] Lugo J, Fadeuilhe C, Gisbert L, et al. Sleep in adults with autism spectrum disorder and attention deficit/hyperactivity disorder: a systematic review and meta-analysis. *Eur Neuropsychopharmacol*. 2020;38:1–24. 10.1016/j.euroneuro.2020.07.004 32712061

[5] Richdale AL, Schreck KA. Sleep problems in autism spectrum disorders: prevalence, nature, & possible biopsychosocial aetiologies. *Sleep Med Rev*. 2009;13(6):403–411. 10.1016/j.smrv.2009.02.003 19398354

[6] Gotham K, Marvin AR, Taylor JL, et al. Characterizing the daily life, needs, and priorities of adults with autism spectrum disorder from interactive autism network data. *Autism.* 2015;19(7):794–804. 10.1177/1362361315583818 PMC4581903 25964655

[7] Singer EV, Niarchou M, Maxwell-Horn A, et al. Characterizing sleep disorders in an autism-specific collection of electronic health records. *Sleep Med*. 2022;92:88–95. 10.1016/j.sleep.2022.03.009 PMC9018608 35367909

[8] Leader G, Barrett A, Ferrari C, et al. Quality of life, gastrointestinal symptoms, sleep problems, social support, and social functioning in adults with autism spectrum disorder. *Res Dev Disabil*. 2021;112:103915. 10.1016/j.ridd.2021.103915 33676088

[9] Morgan B, Nageye F, Masi G, Cortese S. Sleep in adults with autism spectrum disorder: a systematic review and meta-analysis of subjective and objective studies. *Sleep Med*. 2020;65:113–120. 10.1016/j.sleep.2019.07.019 31739229

[10] Zeidan J, Fombonne E, Scorah J, et al. Global prevalence of autism: a systematic review update. *Autism Res*. 2022;15(5):778–790. 10.1002/aur.2696 PMC9310578 35238171

[11] American Psychiatric Association . Diagnostic and Statistical Manual of Mental Disorders. 5th ed. Washington, DC: American Psychiatric Association; 2013.

[12] den Houting J . Neurodiversity: An insider’s perspective. *Autism.* 2018;23(2):271–273. 10.1177/1362361318820762 30556743

[13] McLean KJ, Eack SM, Bishop L. The impact of sleep quality on quality of life for autistic adults. *Res Autism Spectr Disord*. 2021;88:101849. 10.1016/j.rasd.2021.101849 PMC8442542 34539812

[14] Deserno MK, Borsboom D, Begeer S, Agelink van Rentergem JA, Mataw K, Geurts HM. Sleep determines quality of life in autistic adults: a longitudinal study. *Autism Res*. 2019;12(5):794–801. 10.1002/aur.2103 PMC6593837 30969026

[15] Lampinen LA, Zheng S, Taylor JL, et al. Patterns of sleep disturbances and associations with depressive symptoms in autistic young adults. *Autism Res*. 2022;15(11):2126–2137. 10.1002/aur.2812 PMC9677950 36082844

[16] Al-Srehan H, Ayasrah Mohammad N, Al-Rousan Ayoub H, Khasawneh Mohamad Ahmad S, Gharaibeh M. Predicting suicidal ideation among youths with autism spectrum disorder: An advanced machine learning study. *Clin Psychol Psychother*. 2025;32(3):e70082. 10.1002/cpp.70082 40369905

[17] Han GT, Trevisan DA, Abel EA, et al. Associations between sleep problems and domains relevant to daytime functioning and clinical symptomatology in autism: a meta-analysis. *Autism Res*. 2022;15(7):1249–1260. 10.1002/aur.2758 35635067

[18] Benevides TW, Shore SM, Palmer K, et al. Listening to the autistic voice: mental health priorities to guide research and practice in autism from a stakeholder-driven project. *Autism.* 2020;24(4):822–833. 10.1177/1362361320908410 PMC7787673 32429818

[19] Carmassi C, Palagini L, Caruso D, et al. Systematic review of sleep disturbances and circadian sleep desynchronization in autism spectrum disorder: toward an integrative model of a self-reinforcing loop. *Front Psych*. 2019;10:366. 10.3389/fpsyt.2019.00366 PMC6581070 31244687

[20] Verhoeff ME, Blanken LME, Kocevska D, et al. The bidirectional association between sleep problems and autism spectrum disorder: a population-based cohort study. *Mol Autism*. 2018;9(1):8. 10.1186/s13229-018-0194-8 PMC5791216 29423134

[21] Spielman AJ, Caruso LS, Glovinsky PB. A behavioral perspective on insomnia treatment. *Psychiatr Clin N Am*. 1987;10(4):541–553. 10.1016/S0193-953X(18)30532-X 3332317

[22] Lane SJ, Leão MA, Spielmann V. Sleep, sensory integration/processing, and autism: a scoping review. *Front Psychol*. 2022;13:877527. 10.3389/fpsyg.2022.877527 PMC9152214 35656493

[23] Hohn VD, de Veld DMJ, Mataw KJS, van Someren EJW, Begeer S. Insomnia severity in adults with autism spectrum disorder is associated with sensory hyper-reactivity and social skill impairment. *J Autism Dev Disord*. 2019;49(5):2146–2155. 10.1007/s10803-019-03891-8 PMC6483941 30737588

[24] Hirvikoski T, Blomqvist M. High self-perceived stress and poor coping in intellectually able adults with autism spectrum disorder. *Autism.* 2015;19(6):752–757. 10.1177/1362361314543530 25073750

[25] McQuaid GA, Weiss CH, Said AJ, Pelphrey KA, Lee NR, Wallace GL. Increased perceived stress is negatively associated with activities of daily living and subjective quality of life in younger, middle, and older autistic adults. *Autism Res*. 2022;15(8):1535–1549. 10.1002/aur.2779 PMC9545671 35790082

[26] van der Linden K, Simons C, van Amelsvoort T, Marcelis M. Emotional stress, cortisol response, and cortisol rhythm in autism spectrum disorders: a systematic review. *Res Autism Spectr Disord*. 2022;98:102039. 10.1016/j.rasd.2022.102039

[27] Charlton RA, McQuaid GA, Bishop L, Lee NR, Wallace GL. Predictors of sleep quality for autistic people across adulthood. *Autism Res*. 2023;16(4):757–771. 10.1002/aur.2891 36639914

[28] Jovevska S, Richdale AL, Lawson LP, Uljarević M, Arnold SRC, Trollor JN. Sleep quality in autism from adolescence to old age. *Autism Adulthood*. 2020;2(2):152–162. 10.1089/aut.2019.0034 PMC8992849 36601570

[29] Petti T, Gupta M, Fradkin Y, Gupta N. Management of sleep disorders in autism spectrum disorder with co-occurring attention-deficit hyperactivity disorder: update for clinicians. *BJPsych Open*. 2023;10(1):e11. 10.1192/bjo.2023.589 PMC10755553 38088185

[30] Macchia A, Albantakis L, Zebhauser PT, Brandi M-L, Schilbach L, Brem A-K. Autistic adults avoid unpredictability in decision-making. *J Autism Dev Disord*. 2024;55(12):4234–4246. 10.1007/s10803-024-06503-2 PMC12589206 39158770

[31] Recio P, Pozo P, García-López C, Sarriá E. Autistic sensory traits and psychological distress: mediating role of worry and intolerance of uncertainty. *Brain Sci*. 2024;14(11):1088. 10.3390/brainsci14111088 PMC11591926 39595851

[32] Baker EK, Richdale AL, Hazi A, Prendergast LA. Assessing a hyperarousal hypothesis of insomnia in adults with autism spectrum disorder. *Autism Res*. 2019;12(6):897–910. 10.1002/aur.2094 30896090

[33] Richdale AL, Baker E, Short M, Gradisar M. The role of insomnia, pre-sleep arousal and psychopathology symptoms in daytime impairment in adolescents with high-functioning autism spectrum disorder. *Sleep Med*. 2014;15(9):1082–1088. 10.1016/j.sleep.2014.05.005 24974199

[34] Baker EK, Richdale AL, Hazi A. Employment status is related to sleep problems in adults with autism spectrum disorder and no comorbid intellectual impairment. *Autism.* 2019;23(2):531–536. 10.1177/1362361317745857 29455555

[35] Riemann D, Espie CA, Altena E, et al. The European Insomnia Guideline: An update on the diagnosis and treatment of insomnia 2023. *J Sleep Res*. 2023;32(6):e14035. 10.1111/jsr.14035 38016484

[36] Furukawa Y, Sakata M, Yamamoto R, et al. Components and delivery formats of cognitive behavioral therapy for chronic insomnia in adults: a systematic review and component network meta-analysis. *JAMA Psychiatry*. 2024;81(4):357–365. 10.1001/jamapsychiatry.2023.5060 PMC10794978 38231522

[37] van der Zweerde T, Bisdounis L, Kyle SD, Lancee J, van Straten A. Cognitive behavioral therapy for insomnia: a meta-analysis of long-term effects in controlled studies. *Sleep Med Rev*. 2019;48:101208. 10.1016/j.smrv.2019.08.002 31491656

[38] van Straten A, van der Zweerde T, Kleiboer A, Cuijpers P, Morin CM, Lancee J. Cognitive and behavioral therapies in the treatment of insomnia: a meta-analysis. *Sleep Med Rev*. 2018;38:3–16. 10.1016/j.smrv.2017.02.001 28392168

[39] Li C, Luo Q, Wu H. Digital therapeutics for insomnia: an umbrella review and meta-meta-analysis. *NPJ Digit Med*. 2025;8(1):554. 10.1038/s41746-025-01946-y PMC12394607 40877427

[40] Hertenstein E, Trinca E, Wunderlin M, et al. Cognitive behavioral therapy for insomnia in patients with mental disorders and comorbid insomnia: a systematic review and meta-analysis. *Sleep Med Rev*. 2022;62:101597. 10.1016/j.smrv.2022.101597 35240417

[41] Pattison E, Papadopoulos N, Marks D, McGillivray J, Rinehart N. Behavioural treatments for sleep problems in children with autism spectrum disorder: a review of the recent literature. *Curr Psychiatry Rep*. 2020;22(9):46. 10.1007/s11920-020-01172-1 32661719

[42] Mijnster T, Boersma GJ, Meijer E, Lancel M. Effectivity of (personalized) cognitive behavioral therapy for insomnia in mental health populations and the elderly: An overview. *J Pers Med*. 2022;12(7):1070. 10.3390/jpm12071070 PMC9319701 35887566

[43] Cullen M, McCrory S, Hooman G, Coyle M, Fleming L. Effectiveness of Cognitive Behavioural Therapy for insomnia (CBT-I) in individuals with neurodevelopmental conditions: a systematic review. *J Sleep Res*. 2025;34(5):e70058. 10.1111/jsr.70058 PMC12426716 40180888

[44] Vargas C, Paoletti D, De Stasio S, Berenguer C. Sleep disturbances in autistic children and adolescents: a systematic review and meta-analysis of randomized controlled trials. *Autism.* 2025;29(7):1661–1673. 10.1177/13623613251319391 39968574

[45] Ishii M, Ito W, Karube Y, et al. Effects of transdiagnostic group treatment for sleep disturbances in adult attention-deficit/hyperactivity disorders and autistic spectrum disorder: a pilot study. *Sleep Biol Rhythms*. 2022;20(2):173–180. 10.1007/s41105-021-00351-8 PMC10899906 38469265

[46] Lawson LP, Richdale AL, Denney K, Morris EMJ. ACT-i, an insomnia intervention for autistic adults: a pilot study. *Behav Cogn Psychother*. 2023;51(2):146–163. 10.1017/s1352465822000571 36537291

[47] De Crescenzo F, D'Alò GL, Ostinelli EG, et al. Comparative effects of pharmacological interventions for the acute and long-term management of insomnia disorder in adults: a systematic review and network meta-analysis. *Lancet.* 2022;400(10347):170–184. 10.1016/S0140-6736(22)00878-9 35843245

[48] Relia S, Ekambaram V. Pharmacological approach to sleep disturbances in autism spectrum disorders with psychiatric comorbidities: a literature review. *Med Sci (Basel)*. 2018;6(4):95. 10.3390/medsci6040095 PMC6313590 30366448

[49] Halstead E, Sullivan E, Zambelli Z, Ellis JG, Dimitriou D. The treatment of sleep problems in autistic adults in the United Kingdom. *Autism.* 2021;25(8):2412–2417. 10.1177/13623613211007226 33966490

[50] Nijhof D, Melville C, Rydzewska E, Pavlopoulou G, Meehan L, Gardani M. Experiences of and treatment preferences for insomnia in autistic adults: An interpretative phenomenological analysis. *Sleep Med*. 2024;122:163–170. 10.1016/j.sleep.2024.08.011 39178754

[51] Doherty M, McCowan S, Shaw SCK. Autistic SPACE: a novel framework for meeting the needs of autistic people in healthcare settings. *Br J Hosp Med*. 2023;84(4):1–9. 10.12968/hmed.2023.0006 37127416

[52] Sehlin H, Hedman Ahlström B, Andersson G, Wentz E. Experiences of an internet-based support and coaching model for adolescents and young adults with ADHD and autism spectrum disorder - a qualitative study. *BMC Psychiatry*. 2018;18(1):15. 10.1186/s12888-018-1599-9 PMC5774035 29347983

[53] Stenberg JH, Ritola V, Joffe G, Saarni S, Rosenström T. Effectiveness of mobile-delivered, therapist-assisted cognitive behavioral therapy for insomnia in nationwide routine clinical care in Finland. *J Clin Sleep Med*. 2022;18(11):2643–2651. 10.5664/jcsm.10186 PMC9622989 35929590

[54] De Bruin EJ, van Steensel FJ, Meijer AM. Cost-effectiveness of group and internet cognitive behavioral therapy for insomnia in adolescents: results from a randomized controlled trial. *Sleep.* 2016;39(8):1571–1581. 10.5665/sleep.6024 PMC4945317 27306272

[55] Fang L, Lyu Z, Ai S, et al. Is cognitive behavioral therapy for insomnia more cost-effective? New-perspective on economic evaluations: a systematic review and meta-analysis. *Sleep.* 2024;47(8):zsae122. 10.1093/sleep/zsae122 38795362

[56] Darden M, Espie CA, Carl JR, et al. Cost-effectiveness of digital cognitive behavioral therapy (Sleepio) for insomnia: a Markov simulation model in the United States. *Sleep.* 2021;44(4):zsaa223. 10.1093/sleep/zsaa223 33151330

[57] Ellison KS, Guidry J, Picou P, Adenuga P, Davis TE 3rd. Telehealth and autism prior to and in the age of COVID-19: a systematic and critical review of the last decade. *Clin Child Fam Psychol Rev*. 2021;24(3):599–630. 10.1007/s10567-021-00358-0 PMC8191715 34114135

[58] Valencia K, Rusu C, Quiñones D, Jamet E. The impact of technology on people with autism spectrum disorder: a systematic literature review. *Sensors (Basel)*. 2019;19(20):4485. 10.3390/s19204485 PMC6832622 31623200

[59] Li Y-F, Byrne S, Yan W, Ewoldt KB. Self-monitoring intervention for adolescents and adults with autism: a research review. *Behav Sci*. 2023;13(2):138. 10.3390/bs13020138 PMC9952041 36829367

[60] Westerberg B, Bäärnhielm S, Giles C, Hylén U, Holländare F, Bejerot S. An internet based intervention for adults with autism spectrum disorder-a qualitative study of participants experiences. *Front Psych*. 2021;12:789663. 10.3389/fpsyt.2021.789663 PMC8729877 35002808

[61] Mason D, Ingham B, Urbanowicz A, et al. A systematic review of what barriers and facilitators prevent and enable physical healthcare services access for autistic adults. *J Autism Dev Disord*. 2019;49(8):3387–3400. 10.1007/s10803-019-04049-2 PMC6647496 31124030

[62] Harris L, Gilmore D, Hanks C, et al. "It was surprisingly equivalent to the appointment I had in person": advantages and disadvantages of synchronous telehealth for delivering primary care for autistic adults. *Autism.* 2022;26(6):1573–1580. 10.1177/13623613211060589 PMC9149139 34847744

[63] Ali D, O'Brien S, Hull L, Kenny L, Mandy W. 'The key to this is not so much the technology. It's the individual who is using the technology': Perspectives on telehealth delivery for autistic adults during the COVID-19 pandemic. *Autism.* 2023;27(2):552–564. 10.1177/13623613221108010 PMC10076158 35791666

[64] Scheeren AM, Crane L, Heyworth M, Pellicano E. Impact of the COVID-19 pandemic on autistic adults: a scoping review. *Curr Dev Disord Rep*. 2023;10(1):92–122. 10.1007/s40474-023-00268-6 PMC9887236 36741810

[65] Sandgreen H, Frederiksen LH, Bilenberg N. Digital interventions for autism spectrum disorder: a meta-analysis. *J Autism Dev Disord*. 2021;51(9):3138–3152. 10.1007/s10803-020-04778-9 33170393

[66] Wang T, Ma Y, Du X, et al. Digital interventions for autism spectrum disorders: a systematic review and meta-analysis. *Pediatr Investig*. 2024;8(3):224–236. 10.1002/ped4.12417 PMC11427904 39347529

[67] Georén L, Jansson-Fröjmark M, Nordenstam L, Andersson G, Olsson NC. Internet-delivered cognitive behavioral therapy for insomnia in youth with autism spectrum disorder: a pilot study. *Internet Interv*. 2022;29:100548. 10.1016/j.invent.2022.100548 PMC9149190 35651733

[68] Spaargaren KL, Begeer SM, Greaves-Lord K, Riper H, van Straten A. Protocol of a randomized controlled trial into guided internet-delivered cognitive behavioral therapy for insomnia in autistic adults (i-Sleep Autism). *Contemp Clin Trials*. 2024;146:107704. 10.1016/j.cct.2024.107704 39357740

[69] Morin CM, Belleville G, Bélanger L, Ivers H. The Insomnia Severity Index: psychometric indicators to detect insomnia cases and evaluate treatment response. *Sleep.* 2011;34(5):601–608. 10.1093/sleep/34.5.601 PMC3079939 21532953

[70] Vohra R, Madhavan S, Sambamoorthi U. Comorbidity prevalence, healthcare utilization, and expenditures of Medicaid enrolled adults with autism spectrum disorders. *Autism.* 2017;21(8):995–1009. 10.1177/1362361316665222 PMC5517354 27875247

[71] Lai MC, Kassee C, Besney R, et al. Prevalence of co-occurring mental health diagnoses in the autism population: a systematic review and meta-analysis. *Lancet Psychiatry*. 2019;6(10):819–829. 10.1016/s2215-0366(19)30289-5 31447415

[72] Gebara MA, Siripong N, DiNapoli EA, et al. Effect of insomnia treatments on depression: a systematic review and meta-analysis. *Depress Anxiety*. 2018;35(8):717–731. 10.1002/da.22776 29782076

[73] Bai N, Cao J, Zhang H, Liu X, Yin M. Digital cognitive behavioural therapy for patients with insomnia and depression: a systematic review and meta-analysis. *J Psychiatr Ment Health Nurs*. 2024;31(4):654–667. 10.1111/jpm.13024 38226714

[74] Lawrence P, Fulbrook P, Somerset S, Schulz P. Motivational interviewing to enhance treatment attendance in mental health settings: a systematic review and meta-analysis. *J Psychiatr Ment Health Nurs*. 2017;24(9-10):699–718. 10.1111/jpm.12420 28816412

[75] van der Hoek H, Peersmann SHM, Maurice-Stam H, et al. The effect of online cognitive behavioral therapy for insomnia in adolescents and young adults after childhood cancer: results from a randomized controlled trial. *Cancer.* 2025;131(6):e35796. 10.1002/cncr.35796 PMC11883199 40045689

[76] van der Zweerde T, van Straten A, Effting M, Kyle SD, Lancee J. Does online insomnia treatment reduce depressive symptoms? A randomized controlled trial in individuals with both insomnia and depressive symptoms. *Psychol Med*. 2019;49(3):501–509. 10.1017/s0033291718001149 PMC6331685 29747706

[77] Van der Zweerde T, Lancee J, Slottje P, Bosmans JE, Van Someren EJW, van Straten A. Nurse-guided internet-delivered cognitive behavioral therapy for insomnia in general practice: results from a pragmatic randomized clinical trial. *Psychother Psychosom*. 2020;89(3):174–184. 10.1159/000505600 32069463

[78] van Straten A, Emmelkamp J, de Wit J, et al. Guided internet-delivered cognitive behavioural treatment for insomnia: a randomized trial. *Psychol Med*. 2014;44(7):1521–1532. 10.1017/s0033291713002249 24001364

[79] Bastien CH, Vallières A, Morin CM. Validation of the Insomnia Severity Index as an outcome measure for insomnia research. *Sleep Med*. 2001;2(4):297–307. 10.1016/S1389-9457(00)00065-4 11438246

[80] Thorndike FP, Ritterband LM, Saylor DK, Magee JC, Gonder-Frederick LA, Morin CM. Validation of the Insomnia Severity Index as a web-based measure. *Behav Sleep Med*. 2011;9(4):216–223. 10.1080/15402002.2011.606766 22003975

[81] Yang M, Morin CM, Schaefer K, Wallenstein GV. Interpreting score differences in the Insomnia Severity Index: using health-related outcomes to define the minimally important difference. *Curr Med Res Opin*. 2009;25(10):2487–2494. 10.1185/03007990903167415 19689221

[82] Jansson-Fröjmark M, Norell-Clarke A. Psychometric properties of the pre-sleep arousal scale in a large community sample. *J Psychosom Res*. 2012;72(2):103–110. 10.1016/j.jpsychores.2011.10.005 22281450

[83] Halstead EJ, Sullivan EC, Dimitriou D. The impact of COVID-19 on sleep in autistic adults: longitudinal comparisons pre and during lockdown. *Front Psych*. 2021;12:708339. 10.3389/fpsyt.2021.708339 PMC8490657 34621194

[84] Zigmond AS, Snaith RP. The hospital anxiety and depression scale. *Acta Psychiatr Scand*. 1983;67(6):361–370. 10.1111/j.1600-0447.1983.tb09716.x 6880820

[85] Uljarević M, Richdale AL, McConachie H, et al. The hospital anxiety and depression scale: factor structure and psychometric properties in older adolescents and young adults with autism spectrum disorder. *Autism Res*. 2018;11(2):258–269. 10.1002/aur.1872 28922575

[86] Mundt JC, Marks IM, Shear MK, Greist JH. The Work and Social Adjustment Scale: a simple measure of impairment in functioning. *Br J Psychiatry*. 2002;180(5):461–464. 10.1192/bjp.180.5.461 11983645

[87] Zahra D, Qureshi A, Henley W, et al. The work and social adjustment scale: reliability, sensitivity and value. *Int J Psychiatry Clin Pract*. 2014;18(2):131–138. 10.3109/13651501.2014.894072 24527886

[88] van Krugten FCW, Busschbach JJV, Versteegh MM, Hakkaart-van Roijen L, Brouwer WBF. The Mental Health Quality of Life Questionnaire (MHQoL): development and first psychometric evaluation of a new measure to assess quality of life in people with mental health problems. *Qual Life Res*. 2022;31(2):633–643. 10.1007/s11136-021-02935-w PMC8847188 34241821

[89] Boß L, Lehr D, Reis D, et al. Reliability and validity of assessing user satisfaction with web-based health interventions. *J Med Internet Res*. 2016;18(8):e234. 10.2196/jmir.5952 PMC5023944 27582341

[90] Hatcher RL, Gillaspy JA. Development and validation of a revised short version of the working alliance inventory. *Psychother Res*. 2006;16(1):12–25. 10.1080/10503300500352500

[91] Munder T, Wilmers F, Leonhart R, Linster HW, Barth J. Working Alliance Inventory-Short Revised (WAI-SR): psychometric properties in outpatients and inpatients. *Clin Psychol Psychother*. 2010;17(3):231–239. 10.1002/cpp.658 20013760

[92] Herrero R, Vara MD, Miragall M, et al. Working Alliance Inventory for online interventions-Short form (WAI-TECH-SF): the role of the therapeutic alliance between patient and online program in therapeutic outcomes. *Int J Environ Res Public Health*. 2020;17(17):6169. 10.3390/ijerph17176169 PMC7503297 32854381

[93] Skinner HA . The drug abuse screening test. *Addict Behav*. 1982;7(4):363–371. 10.1016/0306-4603(82)90005-3 7183189

[94] Saunders JB, Aasland OG, Babor TF, de la Fuente JR, Grant M. Development of the Alcohol Use Disorders Identification Test (AUDIT): WHO collaborative project on early detection of persons with harmful alcohol consumption--II. *Addiction.* 1993;88(6):791–804. 10.1111/j.1360-0443.1993.tb02093.x 8329970

[95] Zachariae R, Lyby MS, Ritterband LM, O'Toole MS. Efficacy of internet-delivered cognitive-behavioral therapy for insomnia - a systematic review and meta-analysis of randomized controlled trials. *Sleep Med Rev*. 2016;30:1–10. 10.1016/j.smrv.2015.10.004 26615572

[96] Cuijpers P, Miguel C, Harrer M, Ciharova M, Karyotaki E. The overestimation of the effect sizes of psychotherapies for depression in waitlist controlled trials: a meta-analytic comparison with usual care controlled trials. *Epidemiol Psychiatr Sci*. 2024;33:e56. 10.1017/s2045796024000611 PMC11561681 39501660

[97] Parsons CE, Zachariae R, Landberger C, Young KS. How does cognitive behavioural therapy for insomnia work? A systematic review and meta-analysis of mediators of change. *Clin Psychol Rev*. 2021;86:102027. 10.1016/j.cpr.2021.102027 33887655

[98] Lee S, Oh JW, Park KM, Lee S, Lee E. Digital cognitive behavioral therapy for insomnia on depression and anxiety: a systematic review and meta-analysis. *NPJ Digit Med*. 2023;6(1):52. 10.1038/s41746-023-00800-3 PMC10039857 36966184

[99] Lin W, Li N, Yang L, Zhang Y. The efficacy of digital cognitive behavioral therapy for insomnia and depression: a systematic review and meta-analysis of randomized controlled trials. *PeerJ.* 2023;11:e16137. 10.7717/peerj.16137 PMC10624170 37927792

[100] Alimoradi Z, Jafari E, Broström A, et al. Effects of cognitive behavioral therapy for insomnia (CBT-I) on quality of life: a systematic review and meta-analysis. *Sleep Med Rev*. 2022;64:101646. 10.1016/j.smrv.2022.101646 35653951

[101] Cuijpers P, Karyotaki E, Ciharova M, Miguel C, Noma H, Furukawa TA. The effects of psychotherapies for depression on response, remission, reliable change, and deterioration: a meta-analysis. *Acta Psychiatr Scand*. 2021;144(3):288–299. 10.1111/acps.13335 PMC8457213 34107050

[102] Takano Y, Okajima I, Osao M, et al. Remission, treatment response, and treatment adherence across the delivery methods of cognitive behavioral therapy for insomnia: a systematic review and meta-analysis of randomized controlled trials. *Sleep Med Rev*. 2025;84:102204. 10.1016/j.smrv.2025.102204 41259947

[103] Clara MI, van Straten A, Savard J, Canavarro MC, Allen Gomes A. Web-based cognitive-behavioral therapy for insomnia in cancer survivors: the OncoSleep randomized trial. *Sleep Med*. 2025;129:67–74. 10.1016/j.sleep.2025.02.021 39987779

[104] Lin C, Cheng C, Xia Y, et al. Internet-delivered cognitive behavioral therapy for insomnia: the future of insomnia treatment with large language models. *Sleep Med X*. 2025;10:100157. 10.1016/j.sleepx.2025.100157 PMC12615300 41246294

[105] Grist R, Cavanagh K. Computerised cognitive behavioural therapy for common mental health disorders, what works, for whom under what circumstances? A systematic review and meta-analysis. *J Contemp Psychother*. 2013;43(4):243–251. 10.1007/s10879-013-9243-y

[106] Barak A, Hen L, Boniel-Nissim M, Na S. A comprehensive review and a meta-analysis of the effectiveness of internet-based psychotherapeutic interventions. *J Technol Hum Serv*. 2008;26(2-4):109–160. 10.1080/15228830802094429

[107] Maurer JJ, Choi A, An I, Sathi N, Chung S. Sleep disturbances in autism spectrum disorder: animal models, neural mechanisms, and therapeutics. *Neurobiol Sleep Circadian Rhythms*. 2023;14:100095. 10.1016/j.nbscr.2023.100095 PMC10176270 37188242

[108] Harvey AG, Dong L, Hein K, et al. A randomized controlled trial of the Transdiagnostic Intervention for Sleep and Circadian Dysfunction (TranS-C) to improve serious mental illness outcomes in a community setting. *J Consult Clin Psychol*. 2021;89(6):537–550. 10.1037/ccp0000650 PMC9377521 34264701

[109] Futenma K, Takaesu Y, Komada Y, et al. Delayed sleep–wake phase disorder and its related sleep behaviors in the young generation. *Rev Front Psychiatry*. 2023;14:1174719. 10.3389/fpsyt.2023.1174719 PMC10235460 37275982

[110] Steinmetz L, Simon L, Feige B, et al. Network meta-analysis examining efficacy of components of cognitive behavioural therapy for insomnia. *Clin Psychol Rev*. 2024;114:102507. 10.1016/j.cpr.2024.102507 39504928

[111] Thakral M, Von Korff M, McCurry SM, Morin CM, Vitiello MV. Changes in dysfunctional beliefs about sleep after cognitive behavioral therapy for insomnia: a systematic literature review and meta-analysis. *Sleep Med Rev*. 2020;49:101230. 10.1016/j.smrv.2019.101230 PMC7012685 31816582

[112] Miller PK, Bowden SL, Dewison N, Ingham B, Thwaites R, Dagnan D. 'Sometimes I'm feeling baffled and they're probably feeling baffled': on the experiences of psychological therapists working with autistic people in a structured primary care service for anxiety disorders and depression. *Autism.* 2025;29(10):2477–2488. 10.1177/13623613251341610 PMC12417598 40401511

[113] Soh HL, Ho RC, Ho CS, Tam WW. Efficacy of digital cognitive behavioural therapy for insomnia: a meta-analysis of randomised controlled trials. *Sleep Med*. 2020;75:315–325. 10.1016/j.sleep.2020.08.020 32950013

[114] Wang X, Cheng S, Xu H. Systematic review and meta-analysis of the relationship between sleep disorders and suicidal behaviour in patients with depression. *BMC Psychiatry*. 2019;19(1):303. 10.1186/s12888-019-2302-5 PMC6798511 31623600

[115] Brown CM, Newell V, Sahin E, Hedley D. Updated systematic review of suicide in autism: 2018–2024. *Curr Dev Disord Rep*. 2024;11(4):225–256. 10.1007/s40474-024-00308-9

