# Supplementary material for: Effectiveness of guided internet-delivered cognitive behavioral therapy for insomnia in autistic adults (i-Sleep Autism): a randomized controlled trial
Source: Sleep. 2026 Jul 19;49(11):zsag199. doi: 10.1093/sleep/zsag199 (PMC13747339; doi:10.1093/sleep/zsag199)
Supplement: Supplementary file 1 [file supplementary_materials_zsag199.zip › Supplements.docx]

**Effectiveness of Guided Internet-delivered Cognitive Behavioral Therapy for Insomnia in Autistic Adults (i-Sleep Autism): a Randomized Controlled Trial**

Kirsten L. Spaargaren^1,2^, Sander Begeer^1,2^, Smiddy Nieuwenhuis^1,3^, Jessie Bradley^4^, Heleen Riper^1,2,5^, Kirstin Greaves-Lord^6,7^ and Annemieke van Straten^1,2^

^1^Department of Clinical, Neuro-, and Developmental Psychology, Vrije Universiteit Amsterdam, Amsterdam, Netherlands

^2^Amsterdam Public Health Research Institute, Mental Health, Amsterdam

^3^LEARN! Research Institute, Vrije Universiteit, Amsterdam, Netherlands

^4^Policy Analysis section of the faculty of Technology, Policy and Management, Delft University of Technology, Delft, Netherlands

^5^Department of Psychiatry, Amsterdam University Medical Centre, Vrije Universiteit, Amsterdam, Netherlands

^6^Department of Psychology, Clinical Psychology and Experimental Psychopathology Unit, University of Groningen, Groningen, Netherlands

^7^Jonx, Department of (Youth) Mental Health and Autism, Autism Team Northern-Netherlands, Lentis Psychiatric Institute, Groningen, Netherlands

**Corresponding author**

# Correspondence concerning this article should be addressed to Kirsten L. Spaargaren, Department of Clinical, Developmental, and Neuropsychology, Vrije Universiteit Amsterdam, Medical Faculty Building, Van der Boechorststraat 7, 1081 BT, Amsterdam, The Netherlands. Email: [k.l.spaargaren@vu.nl](mailto:k.l.spaargaren@vu.nl).

# Supplementary Materials

**Table S1.**

Post-test sleep medication usage

|  | Control | Intervention | Total |
| --- | --- | --- | --- |
| Variable | *n* = 58 | *n* = 64 | *n* = 122 |
| Sleep medication (past two weeks), n (%) | 24 (37.5%) | 19 (32.8%) | 43 (35.2%) |
| Benzodiazepines & Z-drugs  Melatonin receptor agonist  Antipsychotic  Antidepressant  Alternative remedies  Antihistamine  Cannabis/CBD product  Other^a^ | 12 (18.8%)  4 (6.3%)  7 (10.8%)  3 (4.7%)  2 (3.1%)  1 (1.6%)  1 (1.6%)  3 (4.7%) | 14 (24.1%)  4 (6.9%)  4 (6.9%)  3 (5.2%)  0 (0.0%)  1 (1.7%)  0 (0.0%)  1 (1.7%) | 26 (21.3%)  8 (6.6%)  11 (9.0%)  6 (4.9%)  2 (1.6%)  2 (1.6%)  1 (0.8%)  4 (3.3%) |
| Duration of sleep medication use (past two weeks), mean (SD), days | 10.47 (4.23) | 9.76 (5.29) | 10.16 (4.68) |
| Discontinued/initiated sleep medication compared to baseline, n (%) | 10 (15.6%) | 10 (17.9%) | 20 (16.4%) |

*Note.* Valid percentages displayed respective. ^a^Other medication used for sleep: methylphenidate, acetaminophen, topiramate.

**Table S2.**

Overview of autism-related adaptations to the iCBT-I intervention by module and overall

| Module | Identified concerns | Adaptations implemented |
| --- | --- | --- |
| Module 1: Psychoeducation on normal sleep, sleep disorders, and sleep hygiene | - Lack of autism-specific psychoeducation | - Added autism-specific psychoeducation (prevalence, mechanisms, sleep characteristics) - Included guidance on light exposure, including daylight exposure (at least 15-30 minutes before noon) and evening light reduction (e.g., dim, warm lighting and limiting blue/white light at least 1 hour before bedtime) - Added information on sleep aids such as weighted blankets |
|  | - Limited inclusivity (e.g., focus on paid employment) | - Clarified mixed use of identity-first and person-first language as preferences differ between autistic people - Explicitly stated co-development with autistic stakeholders - Replaced restrictive phrasing (e.g., “paid employment”) with broader, more inclusive language (e.g., “daytime activities”) |
|  | - Information-dense module, making it difficult to maintain overview and identify next steps | - Introduced a structured summary exercise with a concrete action plan |
| Module 2: Sleep scheduling, restriction, and stimulus control | - Difficulties recognizing bodily signals (e.g., fatigue), compounded by hyperfocus | - Acknowledged challenges in recognizing fatigue and emphasized the use of fixed bedtimes instead of relying on this recognition - Addressed the role of hyperfocus in delayed bedtimes |
|  | - Practical and financial barriers to modifying the sleep environment | - Provided low-cost and practical alternatives for optimizing the sleep environment (e.g., room dividers in studio apartments) |
| Module 3: Managing rumination and promoting relaxation | - Suboptimal audio quality causing overstimulation and limited variety of relaxation exercises | - Re-recorded Jacobson’s Progressive Muscle Relaxation exercise - Added new audio exercises (breathing, body scan, and bedtime wind-down), developed with input from a panel member who was a certified yoga instructor |
|  | - Need for more processing time and functional role of worry | - Acknowledged that worry may serve a functional role, more clearly differentiating between “helpful” and “unhelpful” worrying - Extended “scheduled worry time” exercise from 15 to 30 minutes to allow for additional processing time |
| Module 4: Cognitive restructuring of maladaptive sleep-related cognitions | - Terminology perceived as judgmental (e.g., “negative thoughts,” “thinking errors”) | - Reframed terminology from “negative/dysfunctional” to “unhelpful” thoughts - Emphasized that thoughts are not right or wrong, but differ in their impact on well-being and shifted focus from correcting “incorrect” thoughts to developing more helpful and supportive alternatives |
|  | - Limited relevance for individuals without sleep-specific dysfunctional cognitions | - Broadened scope of cognitive restructuring exercise to include cognitions related to general daily stressors (e.g. social interactions) |
| Module 5: Summary and relapse prevention | - Difficulty maintaining progress after completing the intervention | - In addition to summarizing the content and asking what exercises were (not) helpful, we added a final exercise in which participants create a personalized, concrete plan to continue applying learned strategies |
| Across modules | - Perfectionism and fear of “doing it wrong” | - Emphasized that exercises and sleep diaries do not need to be completed perfectly and guiding coaches are not here to “judge”, focusing on personal progress instead |
|  | - Need for more autonomy and flexibility | - Explicitly stated that exercises may be skipped if experienced as unhelpful or unpleasant |
|  | - Perceived lack of relatability due to insufficient autism-specific content and non-autistic (case) examples | - Increased relatability by including autism-specific information and replacing generic case examples (videos and written vignettes) with lived-experience narratives, sharing personal experiences and tips (e.g., one case example perceived increased arousal related to the electric toothbrush at night, and opted for non-electric instead); |
|  | - Perceived resistance towards directive instructions and language | - Clarified instructions and made them informative rather than directive (e.g. “it helps to do this, because..” instead of “you should (not) do this” |
|  | - Limited provision of concrete examples hindering participants’ ability to complete exercises | - Provided more concrete examples to guide exercise completion (e.g. in goal setting of module 1, included example goals, such as “at the end of this intervention, I want to have established a one-hour, relaxing wind-down routine with activity X and Y” |
|  | - Mismatch between module images and content (e.g. woman hanging clothes to dry in sunny weather as banner for module 4), leading to confusion | - Replaced and refined module images to better align with module content and improve interpretability |

*Note*. The original i-Sleep intervention was developed for the general population. Adaptations of this version of the intervention (i-Sleep Autism) were informed by autistic panel members, clinicians, and a literature review.

**Table S3.**

Intervention use, adherence, and engagement characteristics by intervention group (*n* = 82)

| **Variable** | **n (%)** | **M (SD)** | **Min** | **Max** |
| --- | --- | --- | --- | --- |
| **Number of completed sessions**  0  1  2  3  4  5 | 15 (18.3)  67 (81.7)  56 (68.3)  48 (58.5)  46 (56.1)  41 (50.0) | -  -  -  -  -  - | -  -  -  -  -  - | -  -  -  -  -  - |
| **Intervention duration & coach response time** |  |  |  |  |
| Average time spent on full intervention of 5 sessions (days) | 41 (50.0) | 40.83 (11.04) | 23 | 72 |
| Average reaction time of coach (working days) | 67 (81.7) | 3.76 (1.55) | 1 | 9.5 |
| Average reaction time of coach (days) | 67 (81.7) | 4.90 (2.22) | 1 | 14 |
| **Effort put into exercises (0-10 scale)**   1. Evaluating and altering sleep hygiene 2. Bedtime routine 3. Consciously dealing with light exposure 4. Taking moments of rest during the day 5. Sleep restriction 6. Fixed bedtimes 7. Get up at night after being awake > 15-30 minutes 8. Stimulus control 9. Exercises to reduce worrying and rumination 10. Changing/nuancing dysfunctional thoughts about sleep | 58 (70.7)  58 (70.7)  58 (70.7)  58 (70.7)  58 (70.7)  58 (70.7)  58 (70.7)  58 (70.7)  58 (70.7)  58 (70.7) | 7.4 (2.52)  7.4 (2.61)  6.6 (3.19)  6.0 (3.09)  5.7 (4.06)  7.2 (3.15)  4.5 (3.89)  7.4 (3.17)  4.6 (3.66)  4.9 (3.49) | 0  0  0  0  0  0  0  0  0  0 | 10  10  10  10  10  10  10  10  10  10 |

*Note.* Scale 0-10, higher scores denoting greater effort. Sleep hygiene: nutrition, exercise, stimulant use, bedroom characteristics/features; Light exposure: more daylight exposure, less screen light exposure in evenings, dimming the lights in the evenings; Stimulus control: Using the bed(room) only for sleep (and sex).

**Figure S1.**

LMM Interaction effects between time, group and age

*Note.* Timepoint 1: Baseline, Timepoint 2: Three weeks, mid-intervention, Timepoint 3: Six weeks, post-intervention

**Figure S2.**

The association between therapeutic alliance and change scores of insomnia severity

*Note.* Change scores (Baseline – Post intervention assessment) for insomnia severity assessed with the Insomnia Severity Index on the Y-axis, Therapeutic alliance assessed with The Working Alliance Inventory – Short Revised. Greater therapeutic alliance is associated with greater reductions insomnia severity.

**Table S4.**

Evaluation of i-Sleep Autism by intervention group participants (n = 82)

| Outcome | n | M (SD) | Min | Max |
| --- | --- | --- | --- | --- |
| CSQ-I Total | 59 | 21.12 (5.18) | 9 | 30 |
| WAI-SR Total  Goal  Task  Bond | 59  59  59  59 | 39.14 (14.38)  12.93 (5.20)  12.14 (5.12)  14.07 (5.04) | 12  4  4  4 | 60  20  20  20 |
| WAI-TECH-SF Total  Goal  Task  Bond | 59  59  59  59 | 49.68 (20.72)  16.44 (7.00)  16.56 (6.93)  16.42 (6.96) | 12  4  4  4 | 84  28  28  28 |

*Note*. CSQ-8 – Client Satisfaction Questionnaire-8, WAI-SR – Working Alliance Inventory – Short Revised, WAI-TECH-SF – Working Alliance Inventory for Online Interventions – Short Form

**Table S5.**

Reasons for Preferring Online Therapy (Ranked by Weighted Scores)

| Rank →  Reason ↓ | 1 | 2 | 3 | 4 | 5 | 6 | 7 | Total weighed score |
| --- | --- | --- | --- | --- | --- | --- | --- | --- |
| It was flexible to schedule, and I could follow it at my own pace. | 23 | 4 | 8 | 3 | 1 | 1 | 0 | 242 |
| I could follow the program from my own location, I had no travel time. | 6 | 7 | 7 | 8 | 10 | 2 | 0 | 185 |
| I was able to process all the information at my own pace and read it again. | 5 | 11 | 4 | 7 | 4 | 9 | 0 | 179 |
| I find this form of therapy less overwhelming than face-to-face therapy. | 3 | 6 | 9 | 7 | 11 | 4 | 0 | 171 |
| This form of therapy is predictable and structured. | 1 | 9 | 5 | 11 | 8 | 5 | 1 | 165 |
| The anonymity and privacy I had with this online program was nice. | 2 | 3 | 6 | 4 | 6 | 18 | 1 | 133 |
| Other reason | 0 | 0 | 1 | 0 | 0 | 1 | 38 | 45 |

*Note.* Total weighted score as calculated by assigning ranks (1-7) to each reason, with rank 1 receiving the highest weight and rank 7 receiving the lowest weight. The weighted score is the sum of the frequency of each rank multiplied by its respective rank value. Higher total scores indicate greater preference for that reason.

**Table S6.**

Reasons for Preferring Face to Face Therapy (Ranked by Weighted Scores)

| Rank →  Reason ↓ | 1 | 2 | 3 | 4 | 5 | Total weighed score |
| --- | --- | --- | --- | --- | --- | --- |
| I missed direct interaction and personal contact with the therapist, which made me feel less connected | 10 | 3 | 4 | 0 | 1 | 75 |
| In face-to-face therapy I could have processed the information better | 1 | 7 | 3 | 6 | 1 | 55 |
| Other reason | 5 | 4 | 0 | 1 | 8 | 51 |
| Face-to-face therapy would give me more focus on the treatment, because I have to leave home for this | 1 | 2 | 7 | 5 | 3 | 47 |
| It was difficult to motivate myself for this online program | 1 | 2 | 4 | 6 | 5 | 42 |

*Note.* Total weighted score as calculated by assigning ranks (1-5) to each reason, with rank 1 receiving the highest weight and rank 5 receiving the lowest weight. The weighted score is the sum of the frequency of each rank multiplied by its respective rank value. Higher total scores indicate greater preference for that reason.
