# Supplementary material for: Effectiveness of guided internet-delivered cognitive behavioral therapy for insomnia in autistic adults (i-Sleep Autism): a randomized controlled trial
Source: Sleep. 2026 Jul 19;49(11):zsag199. doi: 10.1093/sleep/zsag199 (PMC13747339; doi:10.1093/sleep/zsag199)
Supplement: Supplementary file 1 [file supplementary_materials_zsag199.zip › Feedback form.docx]

Dear participant,

Thank you very much for your valuable participation in the insomnia co-creation group, in which we optimize an online course for insomnia (i-Sleep) and better tailor it to the needs of people with autism. Your involvement and experiences are invaluable to us!

The ultimate goal of this course is to have a positive impact on the well-being of people with autism, by improving sleep, and your feedback plays a crucial role in achieving that goal.

In this document, we ask for your honest opinion on the different modules. We like to know what works well and where we can make improvements. Your perspective is unique and valuable, and we hope you'll openly share your thoughts and ideas with us.

Important notes:

- You can take your time to review and review i-Sleep. However, it is not our intention that you invest a lot of time on your own to make the course "perfect". It is the joint input of the co-creation group with which we as researchers will improve the course.
  - Our time indication for this part of the co-creation group is **a maximum of 4 to 5 hours.**
  - After that, we would like to ask you to stop, even if you have not looked at or filled in everything.
  - If you enjoy it and would like to complete the feedback, you are free to do so. Any additional suggestion is of course welcome.
- Would you please return this document to k.l.spaargaren@vu.nl [before Wednesday 13 December](mailto:k.l.spaargaren@vu.nl)?

Once again, thank you for your time and commitment. We greatly appreciate your contributions.

With kind regards,

Kirsten Spaargaren
Prof. Sander Begeer
Prof. Annemieke van Straten

feedback form i-Sleep

Co-creation group insomnia

Content

[Instructions on how to fill out this form	2](#_Toc235025971)

[General feedback	3](#_Toc235025972)

[i-Sleep: Preparation for the module	3](#_Toc235025973)

[i-Sleep Module 1: Sleep and your habits	4](#_Toc235025974)

[i-Sleep Module 2: Improve your sleep patterns	5](#_Toc235025975)

[i-Sleep Module 3: Less worrying, more relaxing!	6](#_Toc235025976)

[i-Sleep Module 4: Changing your thoughts	7](#_Toc235025977)

[i-Sleep Module 5: And now?	8](#_Toc235025978)

# Instructions on how to fill out this form

You will use this form to assess the i-Sleep course.

You only have to rate the modules (sessions), we cannot change the smartphone application for the diary and the chat.

To provide suggestions, use the text fields that you will find under each module in this document. The text fields automatically increase in size as you type more. If necessary, remove unnecessary blank spaces at the bottom of the text field.

You don't have to fill in all the fields, just use the ones you need.

Keep a maximum of about 1 A4/page per module.

In terms of time commitment: keep a maximum of about 4/5 hours for the total feedback. If you are not ready after that, we ask you to stop, because we do not want to ask for more time from you. If you enjoy it and still want to continue, you are of course free to do so.

Good luck and have fun!

Do you have any questions? You can always send an e-mail to k.l.spaargaren@vu.nl.

| Last name | Enter your last name here |
| --- | --- |
| Date | Click here to choose the date |

# General feedback

What grade would you rate the course, on a scale of 1-10? Type your number

What do you think of the course in general?

- Explanation	Make your choice
- Examples	Make your choice
- Case stories	Make your choice
- Exercises	Make your choice
- Working method	Make your choice
- Length/duration	Make your choice

Was the information in the online module new to you? Make your choice

General comments about i-Sleep

# i-Sleep: Preparation for the module

Space for comments/ideas/adjustments to make this page more autism-friendly:

# i-Sleep Module 1: Sleep and your habits

What is 'normal' sleep, and what exactly about your sleep? In this session, you will read more about sleep and take a closer look at your own sleeping habits.

Space for comments/ideas/adjustments to make session 1 more autism-friendly (text box automatically expands as you type more):

# i-Sleep Module 2: Improve your sleep patterns

Users learn to use the bedroom only for sleeping and to limit the time in bed to the average nighttime sleep duration.

Space for comments/ideas/adjustments to make session 2 more autism-friendly (text box automatically expands as you type more):

# i-Sleep Module 3: Less worrying, more relaxing!

Exercises for progressive muscle relaxation and techniques to stop worry (e.g., thought blocking and scheduled worry time) are offered.

Space for comments/ideas/adjustments to make session 3 more autism-friendly (text box automatically expands as you type more):

# i-Sleep Module 4: Changing your thoughts

The basics of cognitive behavioral therapy are explained, and the most common dysfunctional ideas about insomnia are discussed.

Space for comments/ideas/adjustments to make session 4 more autism-friendly (text box automatically expands as you type more):

# i-Sleep Module 5: And now?

The key points are summarized, and a plan for the future is being worked on to maintain the improved sleep habits.

Space for comments/ideas/adjustments to make session 5 more autism-friendly (text box automatically expands as you type more):
